# Understanding domain swapping in the c-Src SH3 domain through hinge-loop mutagenesis

**DOI:** 10.1107/S2059798325006977

**Published:** 2025-08-27

**Authors:** M. Carmen Salinas-Garcia, Marina Plaza-Garrido, Jose C. Martinez, Ana Camara-Artigas

**Affiliations:** a Department of Chemistry and Physics University of Almeria, Agrifood Campus of International Excellence (ceiA3) and CIAMBITAL Carretera de Sacramento s/n 04120Almeria Spain; b Department of Physical Chemistry University of Granada Avenida Fuentenueva s/n 18071Granada Spain; F. Hoffmann-La Roche Ltd, Switzerland

**Keywords:** SH3 domains, 3D domain swapping, hinge loop, open/secondary interface, closed/primary interface

## Abstract

The c-Src SH3 domain is one of the best-characterized modular domains from a biophysical and structural point of view. This SH3 domain displays noncanonical alternative folding, forming 3D domain-swapped oligomers and amyloid fibrils. These features make this small protein an ideal model for studying these phenomena. Residues in the regions that favour unfolding of the monomer and those in the hinge loop have been deeply studied in proteins undergoing 3D domain swapping. To study the role of these residues in the unfolding of the c-Src SH3 domain, we have constructed several chimeric proteins by interchanging residues in the RT and n-Src loops between the c-Src SH3 and Abl SH3 domains. The RT (the region between β1 and β2) and n-Src (the region between β2 and β3) loops create two sides of the shallow hydrophobic groove where proline-rich motif sequences bind to the SH3 domain. In addition to the structural information, we have performed a biophysical characterization of these chimeric constructs. The c-Src SH3 domain bearing the loops of the Abl SH3 shows minor changes in stability. Interestingly, these replacements do not prevent the formation of domain-swapped dimers. However, the interchange of one or two loops within the Abl SH3 domain produces a noticeable reduction in its stability but does not promote the formation of 3D domain-swapped oligomers. Thus, our results indicate that although the composition of the hinge loop is likely to play a role in the interchange of structural elements to form the intertwined dimers, it is not the sole driving force in their formation.

## Abstract 2

The role of the hinge loop and the open interface in the 3D domain swapping of the c-Src SH3 domain is reported.

## 1. Introduction

Three-dimensional domain swapping (3D-DS) is a mechanism by which some proteins can oligomerize by exchanging identical domains or structural elements between monomers (Bennett *et al.*, 1994 ▸). This oligomerization mechanism has been reported for many different proteins, and the exchanged regions are diverse in size, sequence and secondary structure (Liu & Eisenberg, 2002 ▸). Although the functional purpose of forming these oligomers is unclear, 3D-DS has been the focus of interest because it has been proposed as one possible mechanism underlying the appearance of early amyloid fibril aggregates (Liu & Eisenberg, 2002 ▸; Bennett *et al.*, 2006 ▸; Ding *et al.*, 2005 ▸; van der Wel, 2012 ▸). The formation of intertwined dimers, or other higher-order oligomers, by 3D-DS emerges as an alternative way of folding the protomer, which could be driven by lower energy than a single folded chain or by the faster formation of the oligomer under given circumstances (Rousseau *et al.*, 2003 ▸; Moschen & Tollinger, 2014 ▸). The swapped oligomers share the hydrophobic core already present in the monomeric form and, in most cases, the secondary-structure elements are conserved (Yang *et al.*, 2005 ▸). The so-called hinge loop facilitates the exchange of structural elements between protomers, a function typically performed by β-turns that connect the secondary-structure elements of the protein (Liu & Eisenberg, 2002 ▸).

To understand the role of residues contained in the hinge loop in affecting the tendency of a protein to undergo 3D-DS self-association, several proteins that form domain-swapped oligomers have been investigated: p13suc1 (Rousseau *et al.*, 2002 ▸, 2004 ▸), RNases (Mazzarella *et al.*, 1995 ▸; Avitabile *et al.*, 2003 ▸; Picone *et al.*, 2005 ▸; Merlino *et al.*, 2008 ▸), calbindin D9k (Håkansson *et al.*, 2001 ▸), NF-κB (Chirgadze *et al.*, 2004 ▸), cytochromes (Hirota *et al.*, 2021 ▸), myoglobin (Xie *et al.*, 2021 ▸), cyanovirin-N (Koharudin *et al.*, 2013 ▸), doublecortin Ub-like domain (Rufer *et al.*, 2018 ▸), catechol-*O*-methyltransferase (COMT; Ehler *et al.*, 2014 ▸) and the Mog1p/PsbP-like fold (May & Gonske, 2025 ▸). The hinge-loop region undergoes the most significant conformational changes, but differences in the number of hydrogen bonds formed upon the opening of the protomer might favour the monomeric or oligomeric form (Xie *et al.*, 2021 ▸). In this way, the domain-swapped oligomer can be stabilized in some cases by forming an antiparallel β-sheet between the protomer chains in the hinge region (Harkiolaki *et al.*, 2006 ▸). Besides differences in contacts at the closed/primary interface and the open/secondary interface, the newly created contact surfaces in the oligomer are also crucial for governing the 3D-DS (Gronenborn, 2013 ▸; Bennett *et al.*, 1994 ▸, 1995 ▸; Gotte *et al.*, 2006 ▸; Noro *et al.*, 2023 ▸).

The Src homology 3 (SH3) domain is a ∼60-residue modular domain that consists of two antiparallel β-sheets orthogonally packed to form a single hydrophobic core. This domain is present in many proteins and regulates critical cellular pathways by recognizing polyproline-rich motifs (PRMs; Kurochkina & Guha, 2013 ▸). The SH3 domains have a degree of sequence and fold similarity, but this similarity is restricted mainly to the β-strand regions of the protein. The five β-strands of the c-Src SH3 domain are connected by loops named RT, n-Src and distal, and the diverging β-turn. The diverse sequence in the β-turns and loops connecting the β-strands, especially the RT (located between the β-strands β1 and β2) and n-Src (located between β-strands β2 and β3) loops, facilitates the specificity and affinity for binding different PRMs. The diverging β-turn is located between β-strands β3 and β4 (Lys103-Lys104-Gly105-Glu106) and does not follow the typical topology of a β-turn, but rather shows an extended and somewhat irregular conformation. Analysis of the ϕ-values suggests that the diverging β-turn and the distal loop (located between β-strands β4 and β5; Ser123-Leu124-Thr125-Thr126) are well ordered in the folding transition state (Grantcharova & Baker, 1997 ▸; Grantcharova *et al.*, 1998 ▸, 2000 ▸; Riddle *et al.*, 1999 ▸; Martinez *et al.*, 1998 ▸; Martinez & Serrano, 1999 ▸). In the folded native state, Ser123 forms a hydrogen bond to Glu106.

Although the typical monomeric β-barrel fold is generally conserved among the SH3 domains, five SH3 domains have been described to fold diversely by forming 3D domain-swapped dimers: Eps8 (Kishan *et al.*, 1997 ▸), p47phox (Yuzawa *et al.*, 2004 ▸), the C-terminal SH3 domain of CRKL (Harkiolaki *et al.*, 2006 ▸), the Nck1-SH3.1 domain (Richter *et al.*, 2020 ▸) and c-Src SH3 (Cámara-Artigas *et al.*, 2009 ▸, 2014 ▸; Bacarizo *et al.*, 2014 ▸). Comparing these intertwined dimers reveals noticeable differences in the hinge loop, while the hydrophobic core of the SH3 domain is mainly conserved. In the Eps8, Nck1 and c-Src SH3 domains, the n-Src loop plays the role of a hinge loop, but their structures show different orientations of the protomers in the intertwined dimer. Meanwhile, in the p47phox SH3 domain, the hinge loop is the distal loop and the intertwined dimer of this domain was present in the context of the whole protein. Finally, the RT loop acts as a hinge loop in the C-terminal SH3 domain of CRKL.

In previous work, the 3D-DS of the c-Src SH3 domain has been characterized and its dependence on experimental conditions has been described (Cámara-Artigas *et al.*, 2009 ▸, 2014 ▸; Bacarizo *et al.*, 2014 ▸). This work explores the role of the hinge-loop composition by interchanging residues in the RT and n-Src loops between two SH3 domains: one being the c-Src SH3 domain, where 3D-DS has been described, and the other being the Abl SH3 domain, where 3D-DS has not been described to date. To understand the principles driving the formation of intertwined structures in the c-Src SH3 domain, we have analysed the effect of these mutations on the opening of the hinge loop and in the primary and secondary interfaces of the oligomers through biophysical characterization and comprehensive structural analysis of chimeric proteins.

## 2. Materials and methods

### 2.1. Cloning, expression and purification of chimeric constructions of the c-Src and Abl SH3 domains

**Figure 1:**
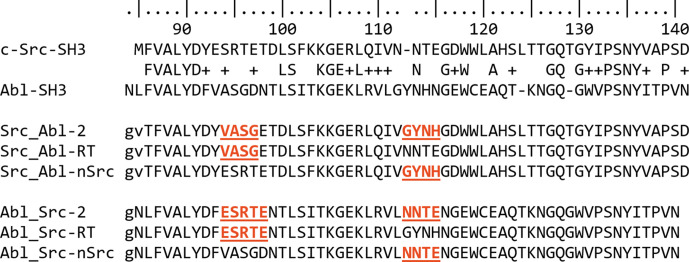
Chimera sequences compared with the WT sequence of the c-Src and Abl SH3 domains. The mutated amino acids are shown in red and underlined.

All genes encoding the protein chimeras described in this work were synthesized by NzyTech (Lisboa, Portugal). Chimeric proteins were constructed by interchanging (i) only the RT loop (Src_Abl-RT and Abl_Src-RT), (ii) only the n-Src loop (Src_Abl-nSrc and Abl_Src-nSrc) or both loops (Src_Abl-2 and Abl_Src-2) (Fig. 1 ▸). Synthetic genes were sub­cloned in the pHTP1 expression vector, including an N-terminal 6×His-tag followed by an engineered TEV cleavage site to eliminate the histidine tag after purification (Sequeira *et al.*, 2017 ▸). The protein was produced in the *Escherichia coli* BL21(DE3) strain and purification was initially carried out following a standard protocol using an Ni^2+^–nitrilotriacetic acid (NTA) column (Takara Bio Europe), as described previously (Bacarizo *et al.*, 2014 ▸). The protein was purified by size-exclusion chromatography using a Superdex 75 16/600 column connected to an ÄKTA FPLC system (GE Healthcare Life Sciences, Barcelona, Spain) equilibrated in 50 m*M* sodium phosphate, 300 m*M* sodium chloride pH 8.0 at a flow rate of 1 ml min^−1^. All of the proteins used in this work showed a single band on SDS–PAGE, with no visible high- or low-molecular-mass contaminants. Pure protein fractions were concentrated to 2–5 mg ml^−1^ in elution buffer and were dialysed against the desired buffer before the experiment. The protein concentration was calculated using the extinction coefficient calculated with the *ProtParam* ExPASy tool (Wilkins *et al.*, 1999 ▸; Gill & von Hippel, 1989 ▸): c-Src SH3 WT, ɛ_280_ = 16 960 *M*^−1^ cm^−1^; Abl-SH3 WT, ɛ_280_ = 15 470 *M*^−1^ cm^−1^; Src_Abl-RT, ɛ_280_ = 16 960 *M*^−1^ cm^−1^; Src_Abl-nSrc, ɛ_280_ = 18 450 *M*^−1^ cm^−1^; Src_Abl-2, ɛ_280_ = 18 450 *M*^−1^ cm^−1^; Abl_Src-RT, ɛ_280_ = 15 470 *M*^−1^ cm^−1^; Abl_Src-nSrc, ɛ_280_ = 13 980 *M*^−1^ cm^−1^; Abl_Src-2, ɛ_280_ = 13 980 *M*^−1^ cm^−1^.

### 2.2. Stability of the Src-Abl and Abl-Src chimeras

#### 2.2.1. Stability versus pH

**Figure 2:**
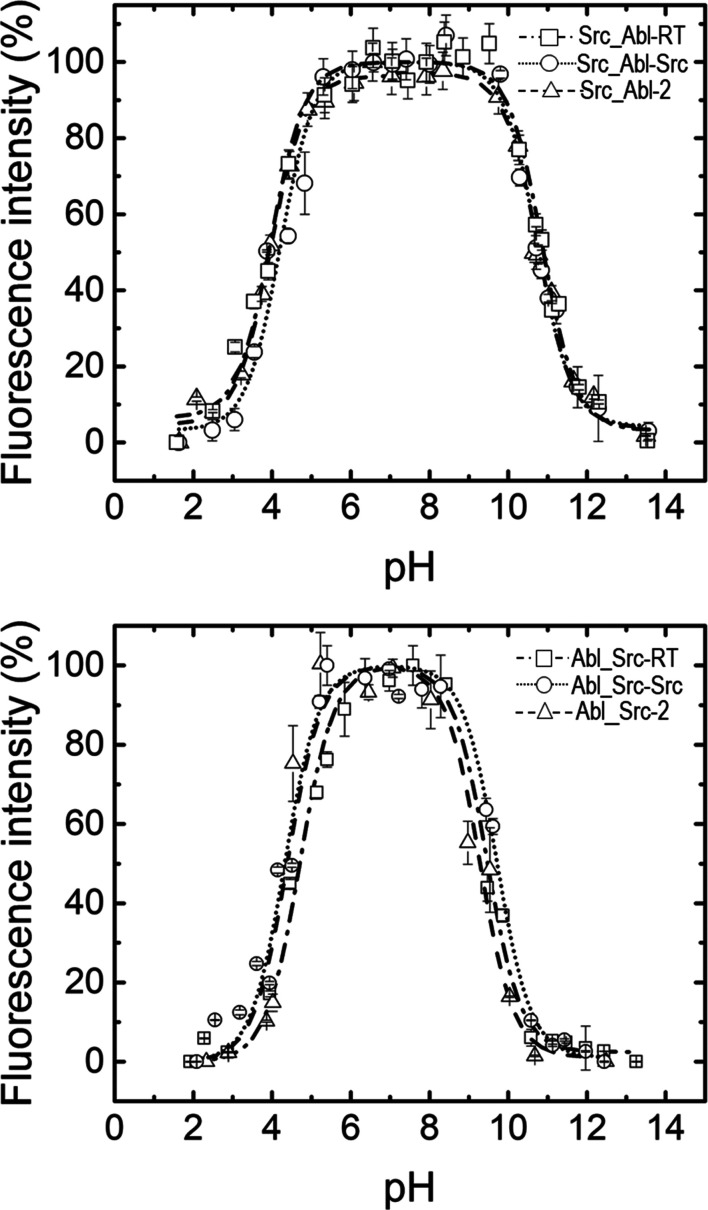
Stability of the chimeras versus pH at 25°C. The samples were excited at 280 nm and the fluorescence intensity was measured at 350 nm. The bandwidth for slits was 5 nm for excitation and emission, and the path length was 1 cm.

The unfolding process of the SH3 domain was followed by measurement of the intrinsic fluorescence of the tryptophan and tyrosine residues. Fluorescence spectra were measured with a PerkinElmer LS-50 spectrofluorimeter. For the pH denaturation experiments, the protein concentration was 2 µ*M* in monomer units. 50 m*M* buffers were prepared using the corresponding salts and acids: pH 2.0–3.0, phosphoric acid/sodium phosphate monobasic; pH 3.0–4.0, formic acid/sodium formate; pH 4.0–5.5, acetic acid/sodium acetate; pH 6.0–7.0, sodium phosphate monobasic/dibasic; pH 7.5–9.0, Tris–HCl; pH 9.5–11.0, carbonic acid/sodium carbonate; pH 11.5–13.0, trisodium phosphate/sodium hydroxide. Appropriate blank corrections were applied to all spectra. At least two independent measurements were conducted for each pH value, and the actual pH value of each sample was measured using a pH meter after the experiment was completed. The samples were excited at 280 nm, and the fluorescence intensity was measured at 350 nm, the maximum of the emission spectra (Supplementary Fig. S1*a*). The bandwidth for slits was 5 nm for excitation and emission, and the path length was 1 cm. The stability of the protein versus pH was analysed at 25°C in the pH range 1–14, as previously reported (Plaza-Garrido *et al.*, 2020 ▸). The apparent p*K*_a_ value of the acid and basic transition can be calculated using equation (1), which is based on the Henderson–Hasselbalch equation (Luiz & Louro, 2011 ▸),
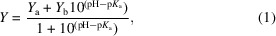
where *Y*_a_ and *Y*_b_ are the intensities of the fractions of the two states in equilibrium (base b and acid a), and the total signal *Y* is a weighted average of the signals of these two species, where the weights are the fractions *Y*_a_ and *Y*_b_ with the boundary condition *Y*_a_ + *Y*_b_ = 1. The parameters in equation (1) were fitted to experimental data using the *Origin* 2019b software (OriginLab, USA). To facilitate comparison among the different pH stability curves in Fig. 2 ▸, the fluorescence intensity was normalized using equation (2),
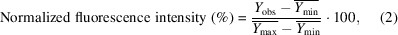
where *Y*_obs_ is the fluorescence intensity measured, 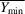 is the average fluorescence intensity of the lower plateau and 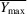 is the average fluorescence intensity of the higher plateau of the stability curve.

#### 2.2.2. Isothermal denaturation by guanidium hydrochloride

The chemical-induced unfolding of the Src and Abl chimeras was measured in the presence of the denaturant guanidine hydrochloride (GdnHCl). Briefly, protein samples at 2 µ*M* in monomer units were prepared in 50 m*M* sodium phosphate pH 7.0 containing different concentrations of GdnHCl and left overnight at 25°C to reach equilibrium. At least two independent measurements were performed for each GdnHCl concentration, and the spectra were all buffer-corrected to eliminate the contribution of GdnHCl to fluorescence. The final GdnHCl concentration was determined using the refractive index using equation (3) (Pace, 1986 ▸),
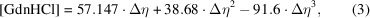
where Δη is the difference in the refractive index of the GdnHCl solution and the buffer.

In these experiments, a displacement of the maximum emission was observed (Supplementary Fig. S1*b*), and the wavelength-averaged emission intensity (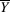), also called the spectrum mass-centre, was calculated using equation (4) (Shirley, 1995 ▸),
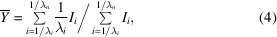
where *I_i_* is the intensity at the wavelength λ_*i*_. From its definition, this parameter is an integral of the value of the fluorescence spectrum, allowing us to obtain information on all of the intensities acquired in the spectrum. The protein unfolding was analysed using the two-state model (Pace & Laurents, 1989 ▸). The value of the Gibbs energy in the absence of denaturant, Δ*G*_w_, was obtained using equation (5) (Bolen & Santoro, 1988 ▸),
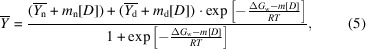
where 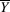 is the wavelength-averaged emission intensity, [*D*] is the denaturant concentration and *m* is the transition slope, a measure of the dependence of the free energy on the denaturant concentration. 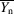 and 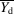 are the wavelength-averaged emission intensity values of the native and unfolded protein, respectively. *m*_n_ and *m*_d_ are the slopes of the native and unfolded protein on the denaturant concentration. The parameters in equation (4) were fitted to experimental data using the *Origin* 2019b software (OriginLab, USA).

The concentration of denaturant at which the protein is half unfolded is given by *D*_1/2_ and can be calculated from equation (6), 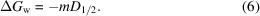
To facilitate comparison among the different denaturation curves, the fraction of folded molecules (*f*_*N*_) was calculated according to equation (7),
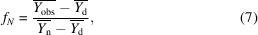
where 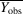 is the spectrum mass-centre calculated from equation (4) for each measurement. 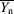 and 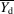 are the averaged spectrum mass-centre values of the native and unfolded protein, respectively.

#### 2.2.3. Thermal denaturation

Thermal unfolding of the Src_Abl and Abl_Src chimeras was determined by circular-dichroism (CD) spectroscopy. CD spectra were recorded at pH 7.0 in 10 m*M* sodium phosphate buffer using a Jasco J-715 spectrophotometer with a coupled Peltier unit. The samples were measured in 0.1 cm path-length quartz cells (Hellma), with a response time of 2 s, a bandwidth of 1 nm and a scan velocity of 50 nm min^−1^ at 25°C. The protein concentration was kept at 20 µ*M* in all experiments. In all measurements, the spectra were corrected by subtracting the baseline. Thermal denaturation was performed using a constant heating rate of 120°C h^−1^ from 10 to 95°C, following the change of ellipticity at 223 nm (Src_Abl mutants) and 230 nm (Abl_Src mutants), based on the maximum difference between the spectrum of the native and unfolded chimeras, while keeping the voltage in a lower noise range (Supplementary Fig S2). The reversibility of the process was verified by comparing the CD spectra of the unheated protein with those obtained from the denatured protein after it was recovered to a temperature of 25°C. Although in some mutants the thermal denaturation was irreversible (see Section 3), an apparent thermal denaturation midpoint, *T*_m_, was obtained to allow a rough estimate of stability based on the change in free energy, Δ*G*. For this purpose, the two-state model represented by equation (5) was converted to changes in temperature instead of the concentration of denaturant agent in equation (8), and the equation was fitted to the change in ellipticity (θ) as a function of temperature,
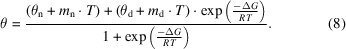
θ_n_ is the ellipticity of the native form and θ_d_ is the ellipticity of the unfolded form. *m*_n_ and *m*_d_ are the slopes of the folded and unfolded regions, respectively. The unfolding Δ*G* is given by equation (9) (Greenfield, 2006 ▸), 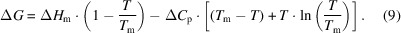
*T*_m_ is the temperature of the midpoint transition between the folded and unfolded form, Δ*C*_p_ is the heat capacity and Δ*H*_m_ is the enthalpy of the unfolding transition. The heat capacity was assumed to be 3.3 kJ K^−1^ mol^−1^ for all of the chimeras based on DSC experiments conducted by Filimonov *et al.* (1999 ▸). It is worth mentioning that the experiments to determine the stability of the chimeras were conducted at a concentration lower than that required to form the oligomers, and the unfolding studies corresponded to the monomeric form of the proteins.

### 2.3. Dynamic light scattering (DLS)

Prior to performing DLS, the solubility of the chimeras was assayed under experimental conditions using the lyophilization method (Trevino *et al.*, 2008 ▸; Supplementary Table S1). DLS experiments were performed in a Zetasizer Nano instrument (Malvern Instruments Ltd, United Kingdom) equipped with a 10 mW helium–neon laser (λ = 632.8 nm) and a thermoelectric temperature controller. The experiments were analysed with *Zetasizer* software (Malvern Instruments Ltd, United Kingdom). Before the measurements, all of the protein samples were centrifuged for 30 min at 14 000 rev min^−1^ (4°C) and filtered through 0.2 µm IC Millex-LG filters (Millipore) to remove any aggregates and dust. Immediately before measurements, the protein solutions were sonicated for 1 min to remove air bubbles.

### 2.4. Crystallization and structure determination

Initial crystallization conditions were determined using the commercial screens Structure Screen 1 and 2 (Molecular Dimensions, UK). All screening experiments were performed using freshly purified protein to maintain the homogeneity of the samples. The best crystallization conditions were optimized and are compiled in Table 1 ▸. The protein concentration ranged from 4 to 10 mg ml^−1^ in 10 m*M* Tris–HCl buffer pH 8.0, except in the crystals where the protein was incubated overnight at 298 K with 5% PEG 300, 10 m*M* acetic/acetate buffer pH 5.0 to favour the formation of intertwined dimers. Some chimeric constructions, such as Src_Abl-nSrc and Abl_Src-nSrc, grew as twinned or tiny crystals and micro-seeding techniques were used to improve the crystal quality (Supplementary Fig. S3). Briefly, the seed stock was prepared from poor-quality crystals, which were crushed using a crystal-crusher tool (Hampton Research, USA). The smashed crystals were aspirated using a pipette and placed into a microcentrifuge tube. To ensure that the crystals were adequately crushed, a seed bead made of steel (Hampton Research, USA) was added to the microcentrifuge tube and the tube was vortexed for 1 min. The crystal solution was centrifuged at 12 000*g* for 5 min. Once the supernatant solution had been removed, the crystal seeds in the pellet were crushed again. Before seeding, the concentrated seed stock was diluted 100 times with 10 m*M* Tris–HCl buffer pH 8.0.

Crystals were harvested from the crystallization drop using Litholoops (Molecular Dimensions, UK) and were quickly vitrified in liquid nitrogen (Pellegrini *et al.*, 2011 ▸). Diffraction data were collected at 100 K on the BL13 XALOC beamline at the ALBA synchrotron, Barcelona, Spain (Juanhuix *et al.*, 2014 ▸) and the ID30A-3 beamline at the ESRF synchrotron, Grenoble, France (McCarthy *et al.*, 2018 ▸). Data were indexed and processed using the *XDS* software (Kabsch, 2010 ▸) and scaled with *AIMLESS* from the *CCP*4 suite (Agirre *et al.*, 2023 ▸) within the *autoPROC* toolbox (Vonrhein *et al.*, 2011 ▸). Data were tested for the possible presence of twinning and pseudo-translation using *phenix.xtriage* (Liebschner *et al.*, 2019 ▸). The data-collection statistics are shown in Table 2 ▸.

All of the structures were solved using the *Phenix* suite (Adams *et al.*, 2010 ▸). Molecular-replacement phasing was performed using *AutoMR* (Afonine *et al.*, 2012 ▸). For the Src_Abl constructions, the coordinates of the monomeric (PDB entry 6xvn) and intertwined dimeric forms (PDB entry 6xvo) of the c-Src SH3 domain (Plaza-Garrido *et al.*, 2020 ▸) were used as a model for molecular replacement. For the Abl_Src constructions, the Abl SH3 domain coordinates were used (PDB entry 3eg3; Palencia *et al.*, 2010 ▸). In some instances, the RT and n-Src loops were removed from the monomeric structure to find a molecular-replacement solution. The final model of each chimeric construction was obtained after several manual building cycles in *Coot* (Emsley & Cowtan, 2004 ▸; Emsley *et al.*, 2010 ▸). Water molecules were automatically modelled using *phenix.refine* in *Phenix* (Afonine *et al.*, 2012 ▸), and manually inspected in the difference electron-density maps. Anisotropic *B* factors were refined for all atoms except water for data at a resolution better than 1.5 Å. At lower resolutions, TLS refinement was applied (Winn *et al.*, 2001 ▸). TLS groups were defined automatically using the *phenix.find_tls_groups* algorithm (Afonine *et al.*, 2012 ▸). The X-ray/geometry weight was determined automatically in each refinement cycle to optimize the balance between the model geometry and the fit to the data.

In the final rounds of refinement, some molecules belonging to the precipitant solution were modelled. The final models were validated using *MolProbity* (Chen *et al.*, 2010 ▸) and *PDB-REDO* (Joosten *et al.*, 2012 ▸). The structure-solution and refinement statistics are shown in Table 3 ▸. Composite omit maps of the hinge regions in the intertwined dimers were obtained using *phenix.composite_omit_map* (Terwilliger *et al.*, 2008 ▸).

### 2.5. Structure analysis

Structure superposition and r.m.s.d. calculations were performed using the *LSQKABCCP*4 module (Kabsch, 1976 ▸). The protein interfaces in the crystal were characterized using the *PISA* server (Krissinel, 2011 ▸). Distances between amino acids were calculated using the *CONTACT* program from the *CCP*4 suite (Krissinel *et al.*, 2022 ▸). Hydrogen-bond and accessible surface area (ASA) analyses were performed with the *VADAR* server (Willard *et al.*, 2003 ▸). The *AlphaFold* server (https://alphafoldserver.com; Abramson *et al.*, 2024 ▸) was used to predict the folding of the chimeras. All structure figures were generated using *PyMOL* 3.1.1 (Schrödinger). The *CASTpFold* server (https://cfold.bme.uic.edu/castpfold/compute) was used to compute interior cavities in the proteins (Ye *et al.*, 2024 ▸).

## 3. Results

### 3.1. Stability of the Src_Abl and Abl_Src chimeras

#### 3.1.1. Stability versus pH

The intrinsic fluorescence of the chimeric proteins was measured over the pH range 1–14 at 25°C to monitor changes in the tertiary structure of the proteins. The normalized fluorescence emission intensity of the Src_Abl (Fig. 2 ▸*a*) and Abl_Src (Fig. 2 ▸*b*) chimeras in the pH range 1–14 shows a bell-shaped dependence with a single transition under acidic and basic pH conditions. The apparent p*K*_a_ values of these transitions calculated with equation (1) are compiled in Table 4 ▸. Although the composition of the loops in the different chimeras introduces differences in ionizable residues with different p*K*_a_ values, only slight changes in the stability range are observed. Src_Abl chimeras are stable between pH 5.0 and 10.0, the same stability range as the WT protein (Plaza-Garrido *et al.*, 2020 ▸). Meanwhile, the Abl_Src chimeras show a shorter stability range between pH 5.5 and 8.5.

#### 3.1.2. Chemical denaturation of the Src_Abl and Abl_Src chimeras

**Figure 3:**
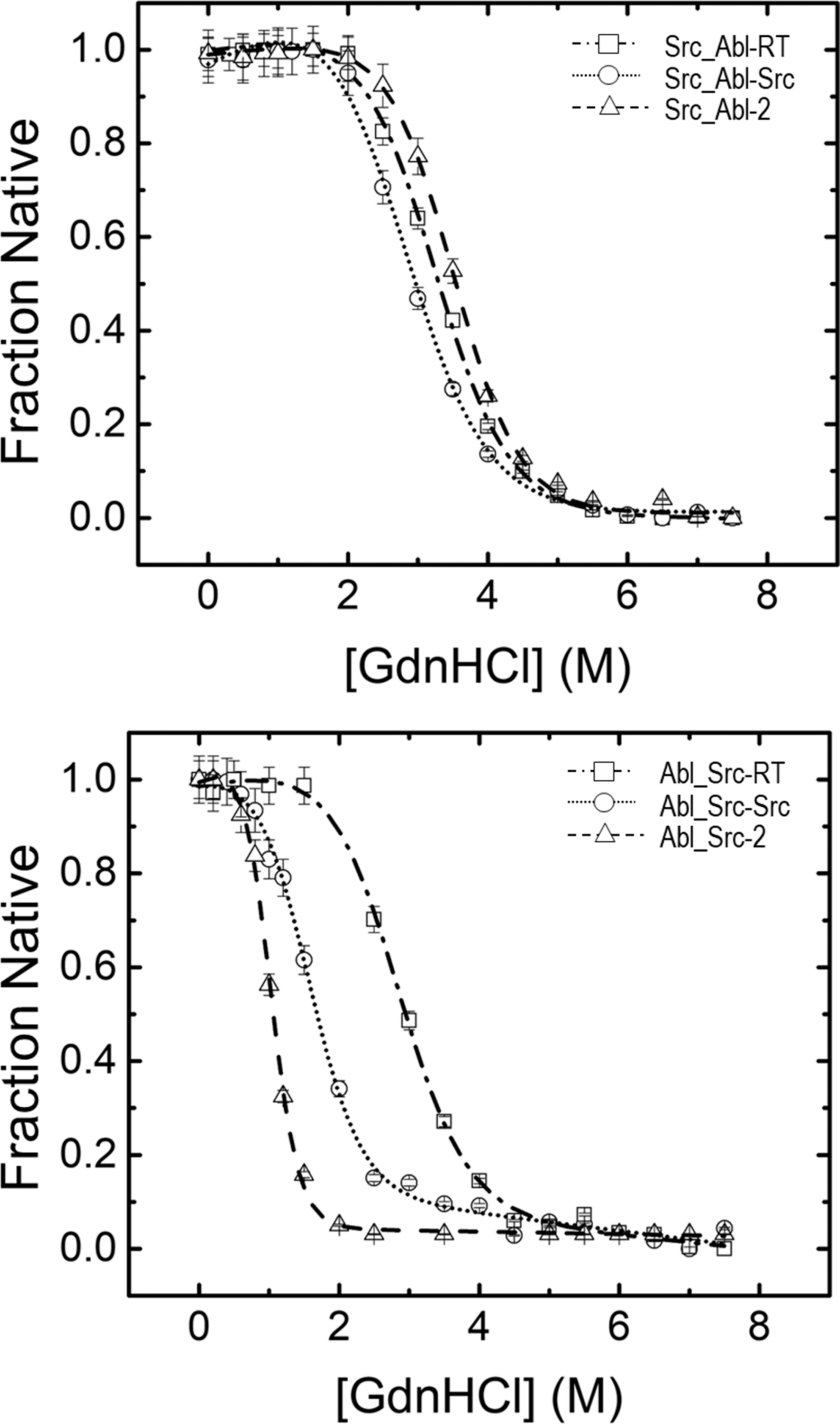
Unfolding of the Src and Abl chimeras in the presence of GdnHCl at 25°C in 50 m*M* sodium phosphate pH 7.0. The protein concentration was 2 µ*M*, and the native fraction is plotted versus GdnHCl concentration. The samples were excited at 280 nm, and the emission spectra were collected between 300 and 500 nm. The bandwidth for slits was 5 nm for excitation and emission, and the path length was 1 cm. The centre of mass was calculated for each spectrum, and the data were normalized to facilitate comparison.

The intrinsic fluorescence of the Src_Abl (Fig. 3 ▸*a*) and Abl_Src (Fig. 3 ▸*b*) chimeras was measured at different concentrations of guanidinium chloride (GdnHCl). The protein unfolding produced by the chemical denaturant was analysed using a two-state model according to equation (5). Table 5 ▸ compiles the thermodynamic parameters. In the Src_Abl chimeras, introducing the residues of Abl SH3 into the RT and n-Src loops barely changes the stability of the protein in the presence of GdnHCl. Conversely, replacing the RT and n-Src loop residues in the Abl_Src chimeras with those from the c-Src SH3 domain results in destabilization under identical denaturing conditions. Notably, the Abl_Src-nSrc and Abl_Src-2 chimeras exhibit pronounced destabilization, highlighting a key role for the c-Src n-Src loop residues in determining the stability of these chimeric proteins.

#### 3.1.3. Thermal denaturation of the Src-Abl and Abl-Src chimeras

The thermodynamic parameters of the thermal denaturations of the chimeras are summarized in Table 6 ▸. All chimeras exhibited reversible denaturation except for Src_Abl-nSrc, which was irreversible. The spectral analysis shows that all of the Src_Abl chimeras have the same *T*_m_ value, which is slightly lower than that of the WT protein. However, the Δ*G* values indicate a decrease in the stability of the chimeras where residues of the n-Src loop of Abl have been introduced. Meanwhile, the Abl_Src chimeras showed lower *T*_m_ values than the WT protein, and the chimeras most affected by the exchange of the loops are those where the n-Src loop of the c-Src was introduced. In this way, the Δ*G* values are the same for the WT and Abl_Src-RT, but the Abl_Src-nSrc and Abl_Src-2 chimeras show a noticeable loss of stability. Similar to the results from chemical denaturation, these data point to lower stability in those chimeras where the n-Src loop was exchanged, whereas the RT loop has a minimal effect on stability.

### 3.2. Crystal structures of the Abl_Src chimeric proteins

**Figure 4:**
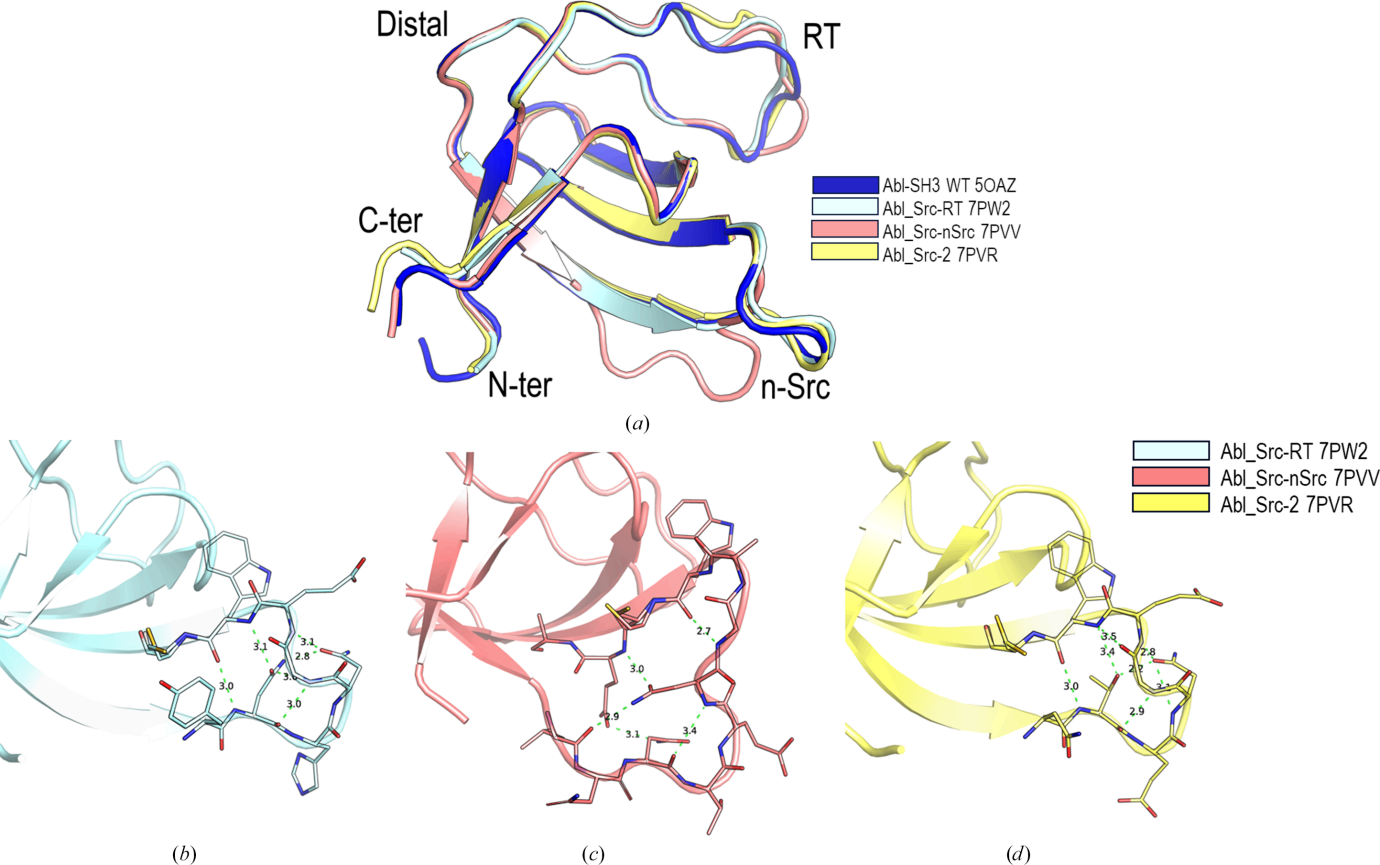
(*a*) Superposition of the Abl SH3 domain (PDB entry 5oaz, blue) and Abl-based chimeric constructions represented as cartoons: Abl_Src-2 (PDB entry 7pvr), Abl_Src-RT (PDB entry 7pw2) and Abl_Src-nSrc (PDB entry 7pvv). (*b*, *c*, *d*) Hydrogen-bond networks in the n-Src loop of (*b*) Abl_Src-2 (PDB entry 7pvr), (*c*) Abl_Src-RT (PDB entry 7pw2) and (*d*) Abl_Src-nSrc (PDB entry 7pvv).

The crystallization conditions of the Abl_Src chimeras have been screened over a broad range of conditions in both the presence and the absence of low-molecular-mass PEGs. The crystal structure of the chimeras shows that substitution of residues at the n-Src and RT loop of the Abl-SH3 domain did not facilitate the formation of intertwined dimers, and all of the Abl_Src chimeras show the same overall fold as the WT variant (PDB entry 5oaz). The Abl_Src-2 crystals were obtained in the absence (PDB entry 7pvq) and the presence (PDB entry 7pvs) of PEG 200, but no differences in the overall fold of this chimera were observed (r.m.s.d. 0.24 Å). Comparison of the Abl_Src-RT (PDB entry 7pw2, r.m.s.d. 0.52 Å) and Abl_Src-2 (PDB entry 7pvs, r.m.s.d. 0.74 Å) chimeras with WT Abl-SH3 shows minor changes in the overall fold (Fig. 4 ▸*a*). However, Abl_Src-nSrc (PDB entry 7pvv, r.m.s.d. 1.22 Å) shows noticeable differences in the conformation of the RT and n-Src loops compared with the WT protein. Although the conformation is slightly different in the RT loop, the hydrogen bonds are conserved. In the n-Src loop, there are significant differences between the WT protein and Abl_Src-nSrc, but also with the Abl_Src-RT and Abl_Src-2 chimeras (Fig. 4 ▸*b*).

### 3.3. Crystal structures of the Src_Abl chimeric proteins

**Figure 5:**
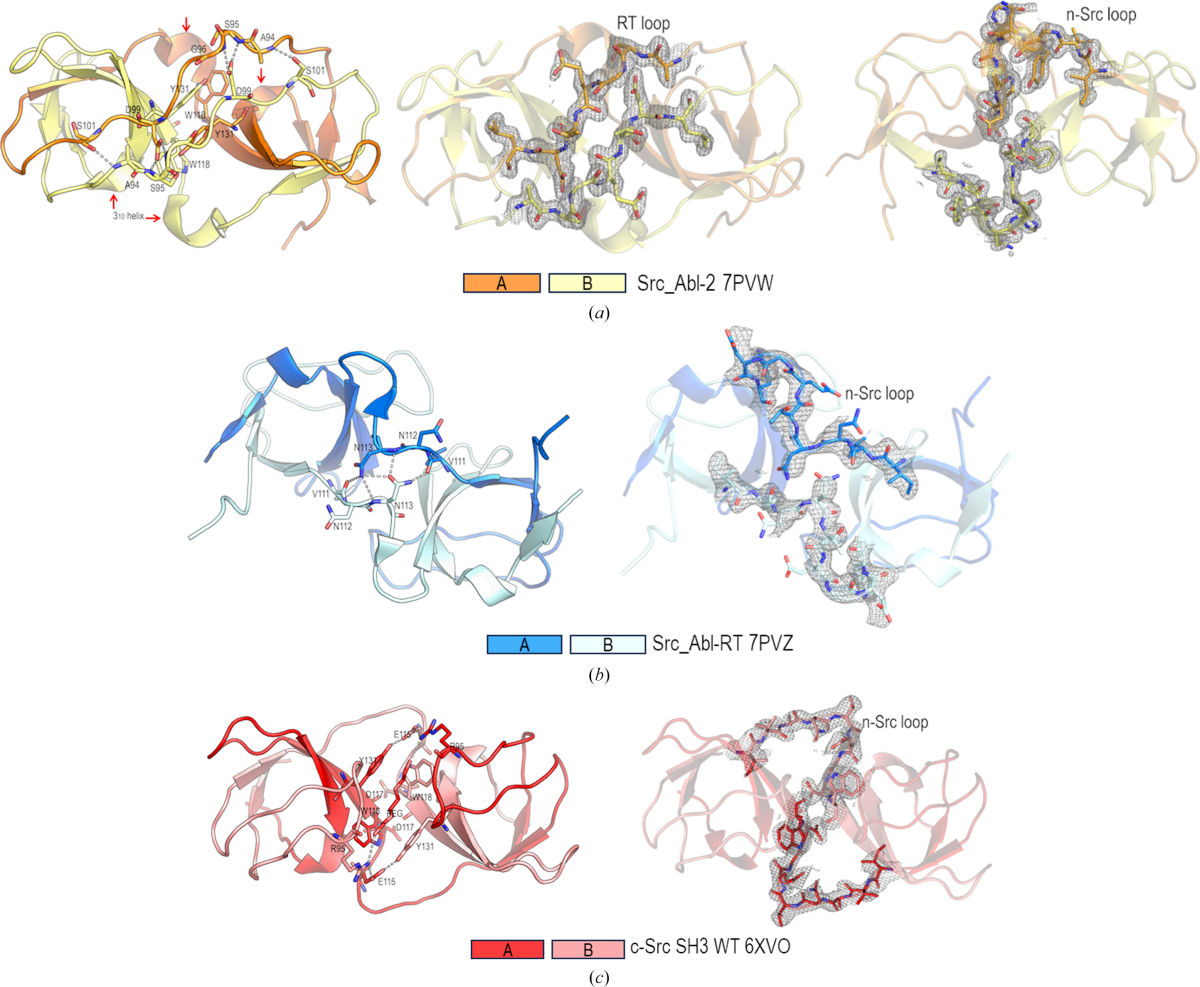
Cartoon representation of the topology of the intertwined dimers of (*a*) Src_Abl-2 (PDB entry 7pvw), (*b*) Src_Abl-RT (PDB entry 7pvz) and (*c*) the WT c-Src SH3 domain (PDB entry 6xvo). Residues involved in new interactions in the open interface are shown as sticks. The new 3_10_-helix in the open interface of Src_Abl-2 is marked with a red arrow. The composite omit map of the residues belonging to the hinge loop in each intertwined dimer is also shown.

No domain swapping has been described in the Abl-SH3 domain. Nevertheless, replacing the residues of the RT and n-Src loops of c-Src with those present in Abl results in the formation of intertwined dimers. Crystallographic structures of the intertwined dimers of the Src_Abl-2 and Src_Abl-RT chimeras were obtained. Interestingly, both domain-swapped structures differ from those previously obtained for the WT c-Src SH3 domain. The Src_Abl-2 dimer (PDB entry 7pvw) forms an intertwined dimer where both RT and n-Src loops act as hinge loops (Fig. 5 ▸*a*). This dimer is present in the solution at acidic pH at 5 mg ml^−1^ concentration (*R*_h_ = 2.4 nm), even in the absence of low-molecular-mass PEGs. At pH 7.0, DLS measurements reveal the presence of a monomer (*R*_h_ = 1.8 nm), even at a concentration higher than 10 mg ml^−1^, the same as the WT protein (Cámara-Artigas *et al.*, 2009 ▸).

In Src_Abl-RT (PDB entry 7pvz), the n-Src loop acts as a hinge loop (Fig. 5 ▸*b*). However, comparison of this dimer with the WT protein shows significant differences (Fig. 5 ▸*c*). DLS experiments show that at pH 5.0 and 7.0, in the absence of PEG 300, this chimera is a monomer in solution (*R*_h_ = 1.8 nm) even at concentrations of >10 mg ml^−1^. However, adding 5% PEG 300 to the protein increases the *R*_h_ value to 2.4 nm at concentrations as low as 5 mg ml^−1^, suggesting the presence of a dimer in the solution. Previously, it has been reported that low-molecular-mass PEG and certain alcohols can induce 3D-DS (Yang *et al.*, 1999 ▸; Hirota *et al.*, 2010 ▸; Wang *et al.*, 2002 ▸). Indeed, the crystal structure of the intertwined dimer of the WT c-Src-SH3 domain was obtained in the presence of 5% PEG. The formation of this intertwined dimer results in a major pocket at the interface between the chains with a volume of 62.473 Å^3^, large enough to accommodate a linear PEG molecule. However, no PEG molecule was found in the open interface of the new intertwined dimers, as is the case with the WT protein.

**Figure 6:**
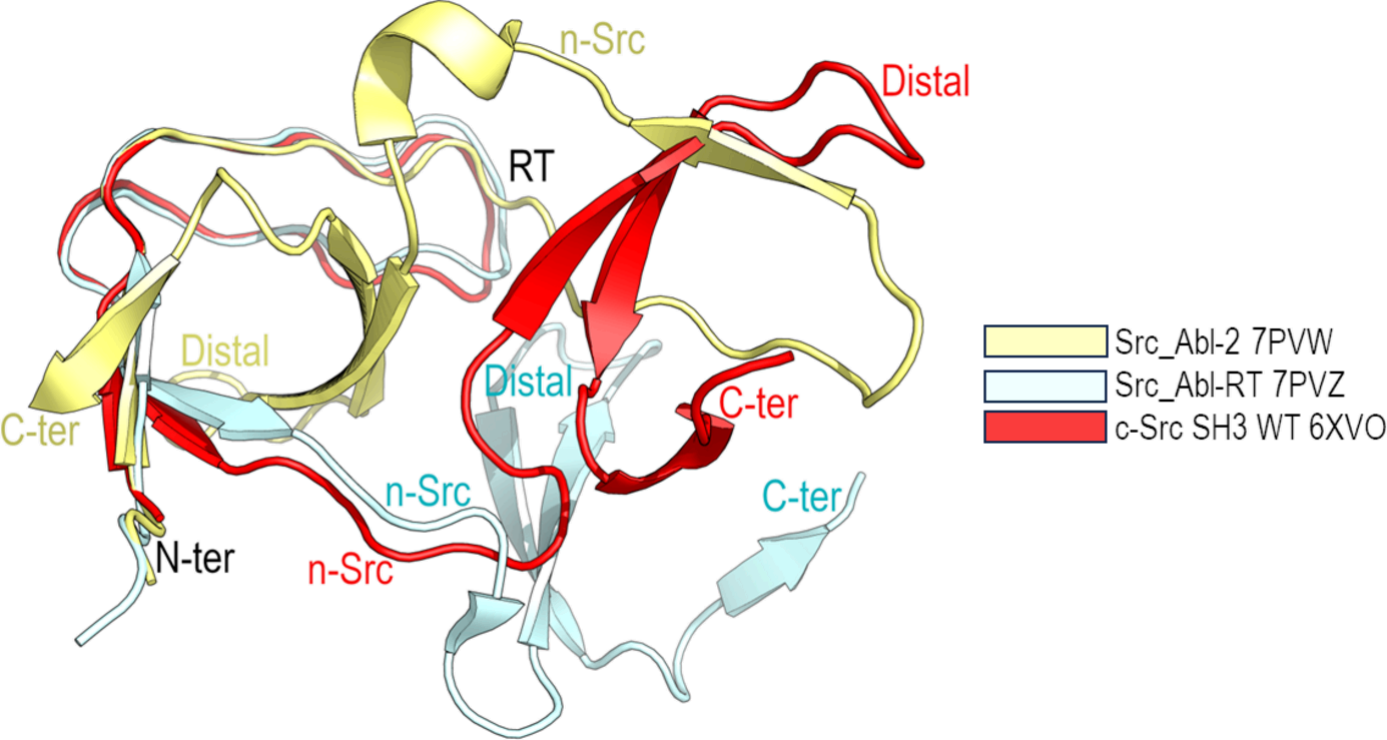
Cartoon representation of the topology of the open monomer of the WT c-Src SH3 domain (PDB entry 6xvo) and the chimeric constructions Src_Abl-2 (PDB entry 7pvw) and Src_Abl-RT (PDB entry 7pvz).

Comparison of the different intertwined dimers reveals the main differences to be in the hinge-loop region, and superposition of the open monomers (excluding the hinge-loop residues) shows a backbone r.m.s.d. value lower than 1 Å. This result points to the conservation of the hydrophobic core of the SH3 domain independently of the protomer orientation or the hinge-loop conformation, sharing the same closed interface (Fig. 6 ▸; Cámara-Artigas *et al.*, 2009 ▸, 2014 ▸; Bacarizo *et al.*, 2014 ▸; Salinas-Garcia *et al.*, 2021 ▸). Thus, the differences between the intertwined dimers of WT c-Src and the Src_Abl chimeras must be found in the open interface of the oligomer.

**Figure 7:**
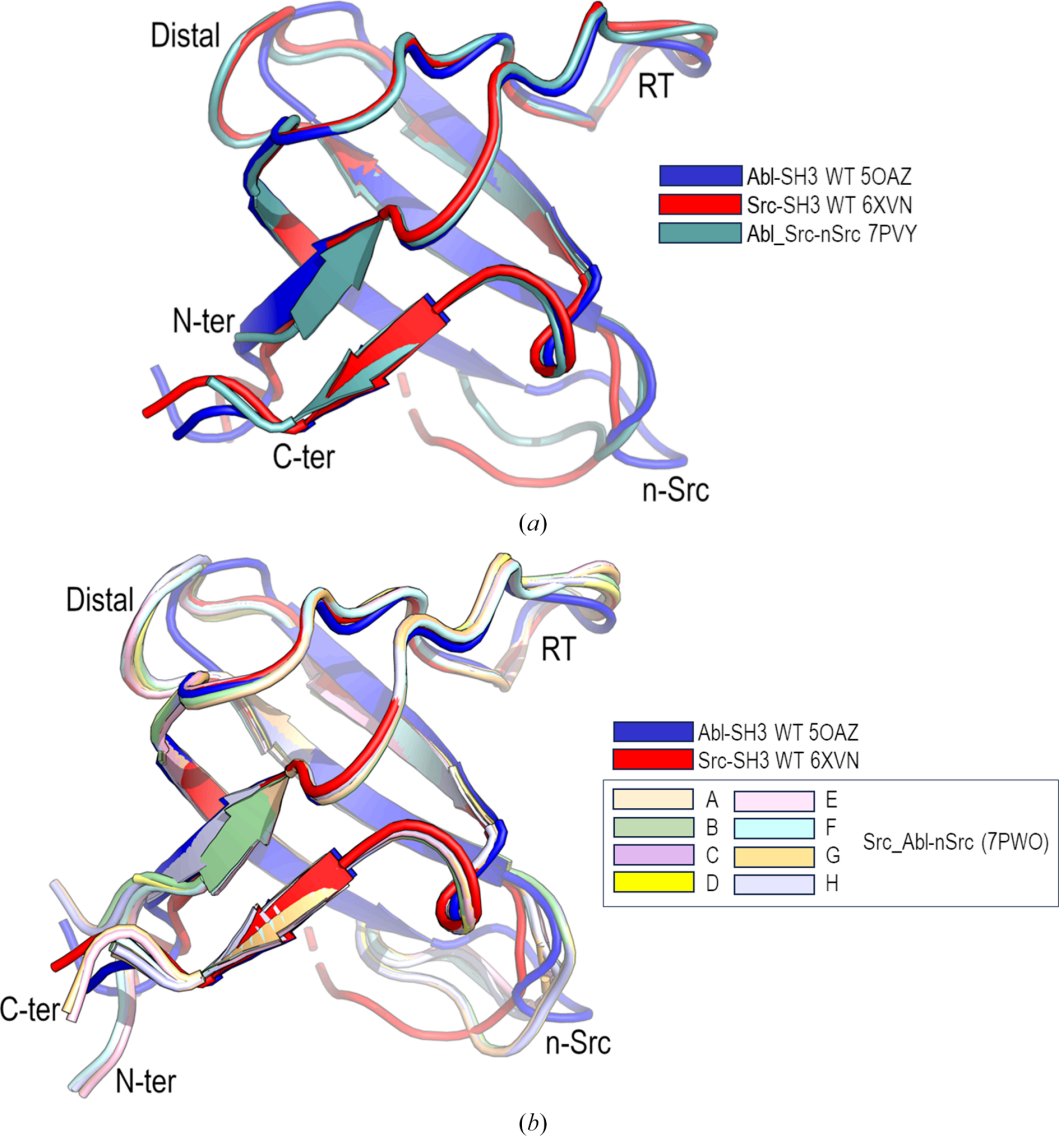
(*a*) Cartoon representation of superposition of the c-Src SH3 domain (PDB entry 6xvn), the Abl SH3 domain (PDB entry 5oaz) and the monomeric form of Src_Abl-RT (PDB entry 7pvy). (*b*) Cartoon representation of superposition of the c-Src SH3 domain (PDB entry 6xvn), the Abl SH3 domain (PDB entry 5oaz) and the eight chains (*A*–*H*) in the Src_Abl-nSrc chimera (PDB entry 7pw0).

The monomeric form of Src_Abl-RT (PDB entry 7pvy) has also been obtained. Comparison with the monomeric structure of the WT protein shows a low r.m.s.d. value (0.70 Å). Interestingly, although the replaced residues were those in the RT loop, the main differences are found in the conformation of the n-Src loop (Fig. 7 ▸*a*), which shows a different conformation from that found in the c-Src SH3 domain, as well as in Abl.

**Figure 8:**
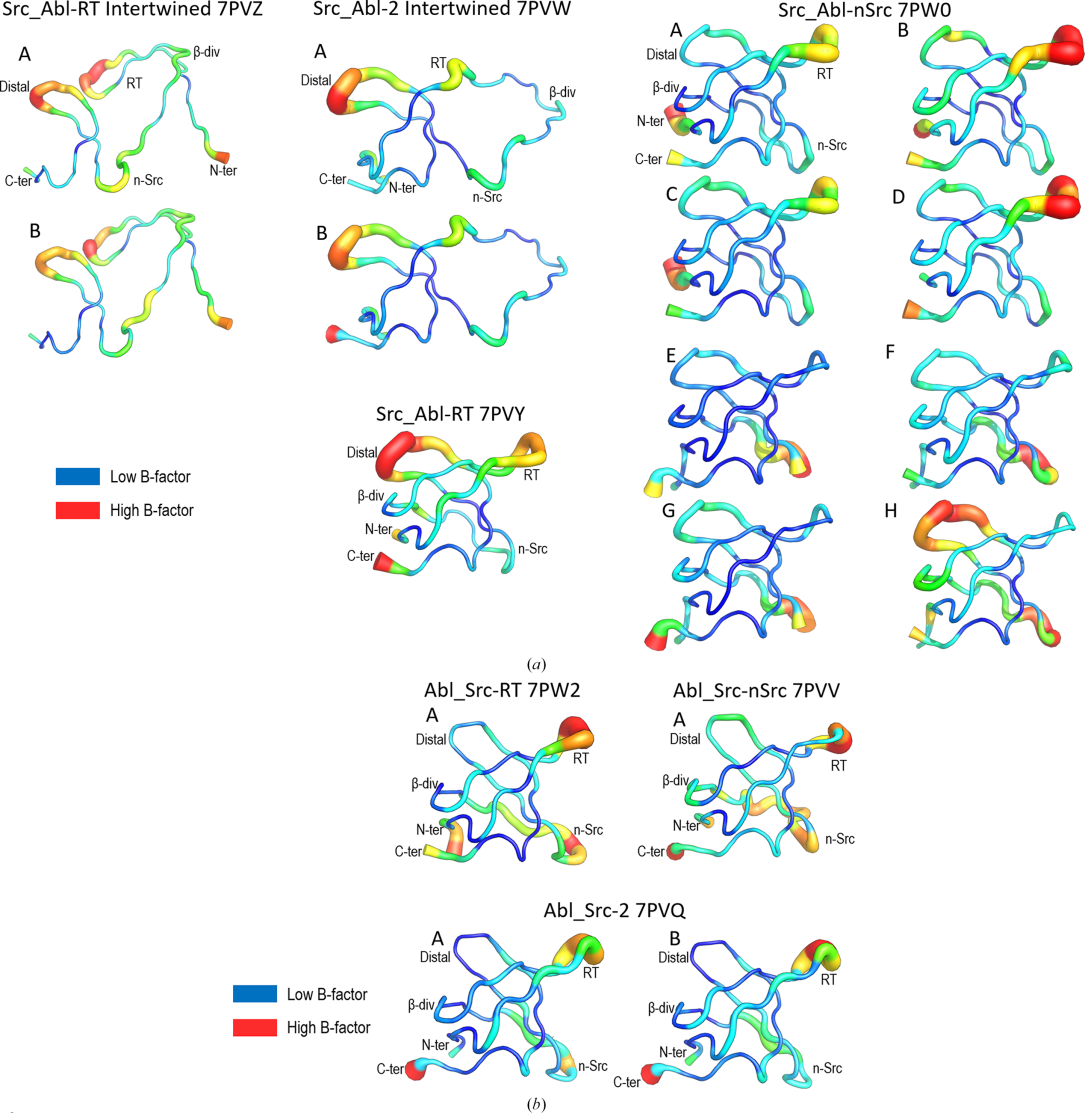
*B*-factor ‘putty’ cartoon representation of the (*a*) Src_Abl and (*b*) Abl_Src chimeras.

Src_Abl-nSrc (PDB entry 7pw0) does not form dimers in all assayed conditions. The structure of this chimera shows eight molecules in the asymmetric unit. It is worth mentioning that the Src_Abl-nSrc chimera has low solubility (∼5 mg ml^−1^ at pH 5.0 and 7.0) compared with the Scr_Abl-RT and Scr_Abl-2 chimeras, and well diffracting crystals were obtained only after applying micro- and macro-seeding techniques in the presence of lithium chloride and glycerol to increase the protein solubility. Comparison of the eight Src_Abl-nSrc molecules in the asymmetric unit only shows minor differences, except in the conformation of the n-Src loop (Fig. 7 ▸*b*). These differences can also be enabled by the presence of crystal contacts. At the same time, these conformations are different from those found in the WT c-Src and Abl SH3 domains. Although introducing residues of Abl into the c-Src SH3 domain is expected to increase the number of hydrogen bonds and reduce loop flexibility in all of the chains, residues in the n-Src loop of some chains show high *B* factors, and the loop has not been fully modelled in two of the eight chains (chain *E* and *H*) present in the asymmetric unit. Interestingly, although the conformation of the RT loop is mainly conserved, residues in this loop show the highest *B* factors in all of the chains (Fig. 8 ▸).

### 3.4. Interactions in the open and closed interfaces of the intertwined dimers

Besides the composition of the hinge loop, the open and closed interfaces of the intertwined structures may also play an important role in forming the domain-swapped structures. The dimeric structures of the Src_Abl-RT and Src_Abl-2 chimeras were compared with those of previous SH3 intertwined dimers to analyse the interactions that facilitate their formation. Indeed, the open interface of the Src_Abl-RT and Src_Abl-2 chimeras differs from that present in WT c-Src SH3. Although all of the chimeras have been obtained in the presence of low-molecular-mass PEGs, this molecule has only been modelled in the interface of the WT dimer (Bacarizo *et al.*, 2014 ▸; Cámara-Artigas *et al.*, 2009 ▸).

As in the WT protein, in the Src_Abl-RT intertwined dimer the n-Src loop is the only hinge loop, although the open protomer shows a different conformation (Supplementary Fig. S4). In the WT protein, a salt bridge between Arg95 and Glu115 stabilizes the dimer. This salt bridge is not present in the Src_Abl-RT (Arg95Ser) and Src_Abl-2 (Arg95Ser and Glu115His) chimeras, where these residues are replaced (Fig. 1 ▸). At the same time, the diverse orientation of the protomers results in new interactions at the open interface. In addition to the salt bridges, hydrogen-bond interactions may facilitate the formation of the dimer. Thus, in the structure of Src_Abl-2, the opening of the RT loop results in the formation of a long β-strand stabilized by several hydrogen bonds: the Ser101A side chain interacts with the backbone N atom of Ala94B, and the Asp99A side chain is hydrogen-bonded to the backbone N atoms of Ser95B and Gly96B (and vice versa) (Fig. 5 ▸*a*). As in the WT, the side chain of Asp117 interacts with the same residue in the complementary chain [Asp117(OD2)A–Asp117(OD2)B, distance 2.5 Å]. Under the crystallization conditions at pH 5.0, the protonated aspartate enables the formation of a hydrogen bond; meanwhile, the anionic form must destabilize the dimer. This might explain why the formation of the intertwined dimer is favoured under mildly acidic pH conditions, where this aspartate residue is protonated (Cámara-Artigas, 2016 ▸; Cámara-Artigas *et al.*, 2009 ▸, 2014 ▸; Bacarizo *et al.*, 2014 ▸). This hypothesis is supported by the crystal structure of the intertwined dimer of the v-Src SH3 W95R-I96T mutant, where the Asp117 residue was replaced by Asn117 (Salinas-Garcia *et al.*, 2021 ▸). The asparagine residue provides a hydrogen bond independently of the pH value, and crystals of the intertwined dimer of the mutant were obtained at higher pH values (pH 6.5). Besides the hydrogen bonds, the burial of the aromatic residues Tyr131 and Trp118 in the open interface must also be considered (Fig. 5 ▸*a*). Besides, in Src_Abl-2 the Abl residues introduced in the n-Src loop form a new 3_10_-helix that results in the shortening of the n-Src loop length and the formation of several hydrogen bonds between the two chains mediated by water molecules.

The analysis of the open interface of the Src_Abl-RT dimer shows hydrogen bonds that differ from those found in the dimers of the Src_Abl-2 and WT protein. In this dimer, the n-Src loop adopts an extended β-strand-like conformation that facilitates the interaction between residues of different chains in the open interface: the Asn113A side chain interacts with the backbone atoms of Val111B and Asn112B and the side chain of Asn113B (and vice versa; Fig. 5 ▸*b*).

## 4. Discussion

### 4.1. Effect of the hinge-loop mutations on 3D-DS

**Figure 9:**
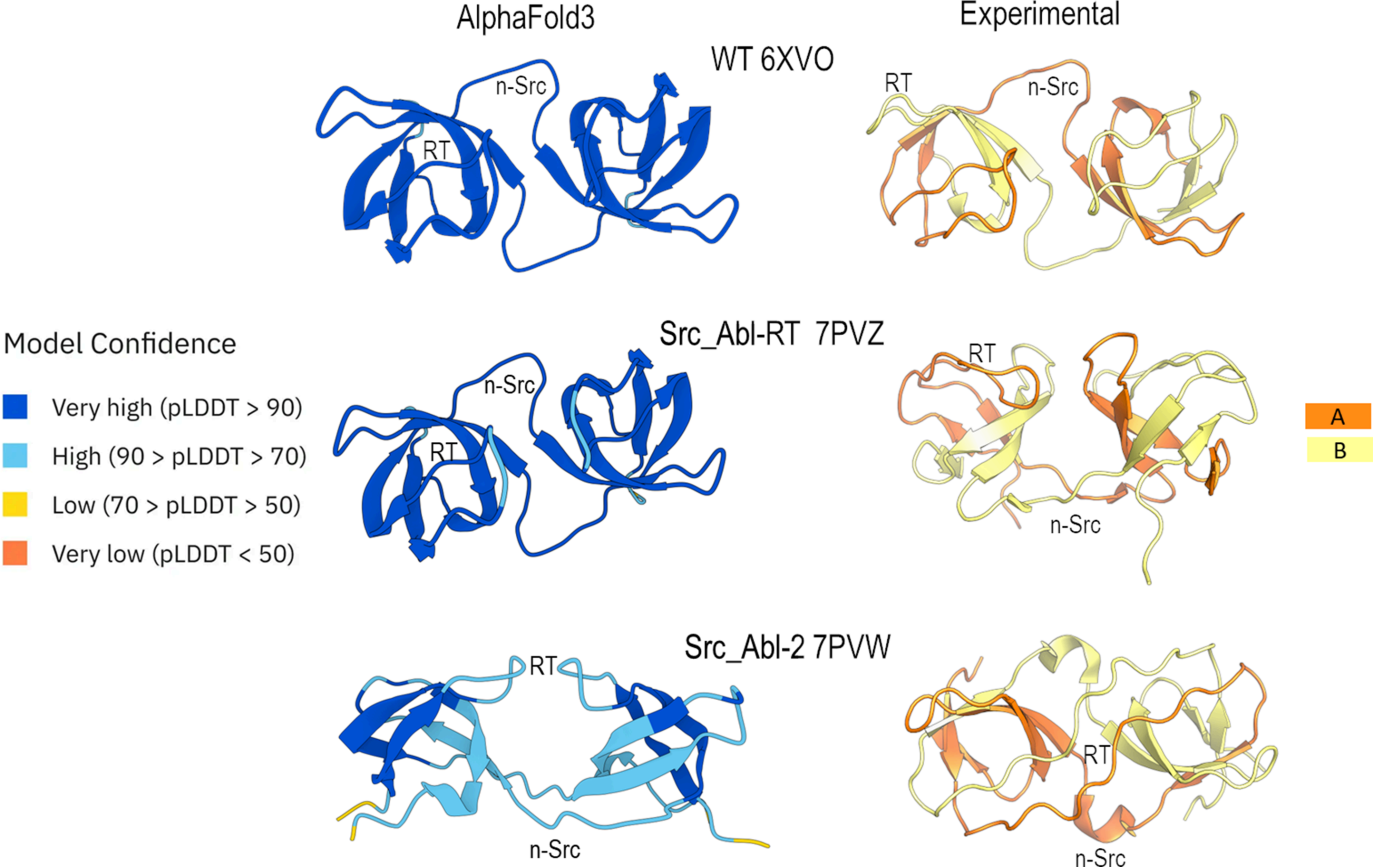
Comparative visualization of the computational models obtained with *AlphaFold*3 and the experimental models of the intertwined dimers of the c-Src SH3 domain. The *AlphaFold*3 models are coloured by the model confidence parameter pLDDT. Chains A and B of the experimental model of the c-Src SH3 domain intertwined dimers are shown.

Since the pioneering work of Anfinsen and coworkers in the 1960s, one of the main goals of scientists has been to predict the three-dimensional structure of a protein just from its sequence (Anfinsen *et al.*, 1961 ▸). In 2021, structural biology witnessed a great revolution with the development of *AlphaFold* (Jumper *et al.*, 2021 ▸). Now, it is possible to predict 3D computational models of proteins just from their sequences using deep-learning algorithms such as *RoseTTAFold*, *AlphaFold*3, *Chai*-1 or *Boltz* (Baek *et al.*, 2021 ▸; Abramson *et al.*, 2024 ▸; Wohlwend *et al.*, 2025 ▸; Boitreaud *et al.*, 2024 ▸). However, it remains an open question whether these algorithms can predict some alternative folding processes, such as 3D-DS or amyloid formation or different conformations (Pinheiro *et al.*, 2021 ▸). In this work, *AlphaFold*3 was used to predict the models of the dimeric constructions. However, the algorithm failed to predict the formation of the new domain-swapped structures (Fig. 9 ▸). Thus, experimental structures are needed to understand the structural determinants governing 3D-DS.

There are several factors to consider when analysing the formation of intertwined structures. An important determinant is the composition of the hinge loop. It determines not only the flexibility of the loop but also the formation of new interactions and secondary-structure elements in the open interface. To form the intertwined dimer, the protein must undergo partial unfolding. Intermediates detected in the folding pathways of small proteins often serve as clues to the folding mechanism, as they possess significant structural information.

Molten globule intermediates are partially folded states that represent a key transitional phase between the completely unfolded and fully folded states of a protein. These transient intermediates have been reported for the urea-induced unfolding of hen egg-white lysozyme by NMR experiments (Schwalbe *et al.*, 1997 ▸). In some proteins, acidic molten globule states can be converted to domain-swapped oligomers. In cytochrome *c* it has been described that molten globule-state oligomers can form 3D-DS oligomers during refolding, where the intermolecular interactions necessary for 3D-DS are already present in the molten globule state. A molten globule state and an on-pathway native-like intermediate have been described for the SH3 domain of PI3 kinase (PIK3; Dasgupta *et al.*, 2014 ▸). There is experimental evidence that supports the idea that most proteins that exhibit 3D-DS are also prone to the formation of amyloids. The c-Src SH3 domain forms both intertwined dimers and amyloid fibrils. However, although the PI3K SH3 domain forms amyloid fibrils and shows folding intermediates, no 3D-DS oligomers have been described to date. It is worth mentioning that although the folding of the c-Src SH3 domain has been thoroughly studied, no intermediates have been described.

An important driving force in 3D-DS is the difference in free energy between the monomer and the swapped oligomer (Liu & Eisenberg, 2002 ▸). Indeed, some molecular-dynamics studies suggest that modifying the hinge loop while maintaining the primary/closed interface is a more effective strategy to promote domain swapping (Woodard *et al.*, 2016 ▸). Besides, residues in the hinge loop can make a difference in protein oligomeric behaviour, as they might be implicated in transient intramolecular interactions during folding, producing traps in the landscape of folding energy, thus increasing the population of intermediates that could favour the formation of domain-swapped oligomers (Gianni *et al.*, 2014 ▸). Considering this approach, we have constructed chimeric proteins in which the RT and n-Src loops of the c-Src and Abl SH3 domains have been interchanged, and we have determined their thermal and chemical stability.

The thermal and chemical stability experiments show that all of the Src_Abl chimeras are slightly less stable than the WT protein. Nevertheless, in the Abl_Src chimeras the loss of stability was larger. Although it has been described that mutations affecting the stability of the protein might facilitate the formation of the intertwined dimer by destabilizing the monomer (Bennett *et al.*, 1995 ▸), our results show a more complex view. Indeed, even though the Abl_Src chimeras are less stable, this reduction in stability does not allow formation of the intertwined dimer. Otherwise, despite the small changes in stability, intertwined dimers of the Src_Abl chimeras have been obtained. In the Abl SH3 domain, the n-Src loop has one additional residue (Fig. 1 ▸) and torsional strain in the hinge loop, generated through the mutation of loop residues and truncation of the loop, can affect the entropic penalty associated with folding (Woodard *et al.*, 2016 ▸; O’Neill *et al.*, 2001 ▸; Liu & Eisenberg, 2002 ▸). The crystal structures of the Abl_Src chimeras show that although there are significant conformational differences in the n-Src loop, several hydrogen bonds facilitate the stiffness of the loop (Fig. 4 ▸*b*). On the contrary, in the c-Src SH3 domain the n-Src loop shows high plasticity, as is manifested by the high *B*-factor values and the difficulties in modelling the whole loop. In those structures where the loop has been fully modelled, only a hydrogen bond between Trp119(NE1) and the backbone carbonyl atoms of residues in the loop is found (Fig. 8 ▸*a*). Introducing the Abl residues into the n-Src loop of the c-Src SH3 domain does not increase hydrogen-bond formation, as happens in the monomeric structures of Src_Abl-nSrc and Src_Abl-RT. Thus, the high flexibility of the hinge loop seems to be essential to loop opening, which facilitates domain swapping in this SH3 domain.

Nevertheless, the results in this work suggest that the hinge loop is not the unique driving force in 3D domain swapping of the c-Src SH3 domain. It has been suggested that because of the solvent exposure of turns and their tolerance to evolutionary variance, they may have little or no effect on the formation of native structures. However, folding studies show that turn residues can affect both protein thermodynamic stability and folding kinetics (Grantcharova *et al.*, 1998 ▸). The interchange between the monomer and the intertwined dimer must occur via an unfolded state, and folding studies can provide insight into the mechanism of this process (Schymkowitz *et al.*, 2000 ▸). Indeed, information about the folding nucleus of the protein can be extracted from measurement of the ϕ-values. The folding of the c-Src SH3 domain has been thoroughly studied by the Baker group (Grantcharova & Baker, 1997 ▸; Grantcharova *et al.*, 1998 ▸, 2000 ▸; Riddle *et al.*, 1999 ▸). These folding studies point to a critical role of the diverging β-turn and the distal loop in the transition state (Grantcharova *et al.*, 1998 ▸). The diverging β-turns of the c-Src and Abl SH3 domain are composed of Lys103-Lys104-Gly105-Glu106 and Thr83-Lys84-Gly85-Glu86, respectively. The differences in the distal loop are noticable, as the c-Src SH3 domain has two additional residues (Ser123-Leu124-Thr125-Thr126-Gly127-Gln128) compared with the Abl SH3 domain (Thr104-Lys105-Asn106-Gly107). A hydrogen bond between Glu106 and Ser123 brings together the diverging β-turn and the distal loop, whereas in the Abl SH3 domain the hydrogen bond is formed by Glu86 and Asn106.

The insertions into the sequence of the distal loop of the c-Src SH3 domain result in a different conformation compared with the Abl SH3 domain, and changes in this loop might be critical for formation of the intertwined dimer. The study of several mutants at position 128 of the c-Src SH3 domain suggests some electrostatic interaction between the residue at position 128 and Glu106 (Bacarizo *et al.*, 2014 ▸). This residue occupies an equivalent position to Asn106 in the Abl SH3 domain, which forms a hydrogen bond with Glu86. Although Gln128 is not in direct contact with Glu106, a cluster of water molecules is placed between them and residues in the distal loop. Additionally, while maintaining the hydrogen bond between Glu106 and Ser123, the distal loop exhibits diverse conformations in the c-Src SH3 domain. As shown in Fig. 8 ▸(*a*), the two chimeras that form an intertwined dimer exhibit the highest *B* factor in the distal loop. We do not have the structure of the monomer of Src_Abl-2, but the monomeric structure of Src_Abl-RT exhibits the same behaviour in the distal loop. As this loop is critical to the folding of the SH3 domain, this feature may explain why the opening of the folded domain is facilitated in these chimeras. However, Src_Abl-nSrc shows an ordered distal loop in most of the molecules in the asymmetric unit and this chimera does not form the intertwined dimer. The same behaviour can be observed in the Abl_Src chimeras, where the distal loop shows low *B* factors (Fig. 8 ▸*b*).

The cartoon putty *B*-factor representation in Fig. 8 ▸ also shows high flexibility in the RT loop in both the Src_Abl and Abl_Src chimeras. This flexibility can also be inferred from the low or negative ϕ-values of the residues at the two ends of the RT loop. Indeed, in the transition state the two terminal strands, the RT loop and the 3_10_-helix are mostly unstructured and contribute few stabilizing interactions (Riddle *et al.*, 1999 ▸). It is worth noting that the flexibility of the RT and n-Src loops has a functional role in the SH3 domains, as these loops create two sides of the shallow hydrophobic groove where proline-rich motif sequences bind to the SH3 domain. These conformational changes are visible when comparing the unbound and proline-rich motif-bound structures (Bacarizo & Camara-Artigas, 2013 ▸; Bacarizo *et al.*, 2015 ▸; Camara-Artigas *et al.*, 2016 ▸; Martin-Garcia *et al.*, 2012 ▸).

Besides the information reported by the *B* factors, which indicate regions with high flexibility or conformational changes, we must also consider these residues that exhibit a non-native conformation in the transition state. Folding studies conducted by Baker and coworkers suggest that Ile110 appears to be critical for the formation of the hydrophobic core during folding. This residue is placed at the beginning of the n-Src loop (residues 110–119: Ile110-Val111-Asn112-Asn113-Thr114-Glu115-Gly116-Asp117-Trp118-Trp119), where the two end residues are part of the hydrophobic core of the SH3 domain. It is worth noting that the two Ile110 mutants (Ile110Val and Ile110Ala) exhibit both slow folding and unfolding rates, suggesting that these mutations destabilize the transition state more than the native or denatured states (Riddle *et al.*, 1999 ▸). In the α-spectrin SH3 domain, Leu33 is located at the same position as the Ile110 residue in the c-Src SH3 domain, and the mutant Leu33Val has an unusual negative ϕ-value pointing to a non-native conformation in the transition state (Martinez & Serrano, 1999 ▸). Interestingly, this residue introduces a break in β-strand β2 of the c-Src SH3 domain (Supplementary Fig. S4).

As the formation of the intertwined dimer is an oligomerization process, another factor to consider is the solubility of the protein. The exchange of loops in the chimeras results in differences in ionizable residues, which might modify the isoelectric point of the protein and its solubility. Although the stability as a function of pH shows minor changes in the Src_Abl and Abl_Src chimeras (Fig. 2 ▸ and Table 4 ▸), these changes modify the p*K*_a_ values of the residues exposed to the solvent, which may affect the solubility of the protein. The Scr_Abl-RT and Scr_Abl-2 chimeras show higher solubility than Scr_Abl-nSrc. Meanwhile, the behaviour is the opposite in the Abl_Src chimeras, and the Abl_Src-n-Src chimera shows higher solubility than the Abl_Src-RT and Scr_Abl-2 chimeras. As the oligomerization process is strongly dependent on protein concentration, differences in solubility might affect the formation of intertwined dimers (Gronenborn, 2013 ▸; Yang *et al.*, 2005 ▸; Kumari & Yadav, 2019 ▸), and the lower solubility of the Scr_Abl-nSrc and Abl_Src chimeras would prevent dimer formation at the maximum attainable concentration (Yang *et al.*, 2005 ▸).

### 4.2. Role of the open interface in 3D-DS

The formation of new interactions in the open interface also contributes to stabilizing the intertwined dimer over the monomer (Xie *et al.*, 2021 ▸). Indeed, the structures of the intertwined dimers of the SH3 domains show new interactions at the open interface, some of which are pH-dependent. Several proteins have been described to form domain-swapped oligomers by altering pH, and most of them undergo domain swapping at acidic pH (Shingate *et al.*, 2015 ▸). It is expected that destabilization of the monomer by the pH would promote the opening of the protomer that, under some circumstances, would form a domain-swapped oligomer. On the contrary, some proteins form stable domain-swapped structures at neutral pH, and their monomeric structures are obtained at acidic pH. The domain-swapped dimer of the Eps8 SH3 domain was obtained at neutral pH (PDB entry 1i07), and the monomeric form was crystallized at pH 4.0 (PDB entry 1i0c) (Kishan *et al.*, 2001 ▸). Another example is the queen bee pheromone binding protein (ASP1), the monomeric structure of which was obtained at acidic pH (PDB entry 3d75) and the intertwined dimer at pH 7.0 (PDB entry 3cz2) (Pesenti *et al.*, 2009 ▸). Interestingly, the single mutation of Asp35Asn in ASP1 disrupts dimer formation, suggesting a significant role of the pH-dependent electrostatic interactions in domain swapping (Shingate *et al.*, 2015 ▸). The effect of electrostatic interactions in dimer formation has also been characterized in the c-Src SH3 domain, where an intertwined dimer was obtained at a mildly acidic pH, and the Asp117Asn mutation in the n-Src loop facilitates the formation of the intertwined dimer at near-neutral pH values (Salinas-Garcia *et al.*, 2021 ▸).

Another factor to consider in 3D-DS of the c-Src SH3 domain and Src_Abl chimeras is the role of low-molecular-mass PEG in domain swapping. As reported in previous work, a high protein concentration promotes 3D-DS and the formation of amyloid fibrils (Plaza-Garrido *et al.*, 2020 ▸; Bacarizo *et al.*, 2014 ▸). The two chimeras forming 3D-DS, Scr_Abl-RT and Scr_Abl-2, also form amyloid-like aggregates at protein concentrations higher than 10 mg ml^−1^ and pH 5.0 (0.1 *M* sodium chloride, 50 m*M* sodium acetate/acetic acid) and 7.0 (0.1 *M* sodium chloride, 50 m*M* sodium phosphate) (*T* = 25 and 37°C; results not shown). Nevertheless, no amyloid formation has been detected for Scr_Abl-nSrc. Nonetheless, adding low-molecular-mass PEG stabilizes the dimer, avoiding amyloid formation. In the intertwined dimer of the WT protein, the contribution to the stability of this crowding agent is evident as a PEG molecule is part of the open interface of the dimer. However, although the intertwined dimers of the Src_Abl-RT and Src_Abl-2 chimeras have been obtained in the presence of low-molecular-mass PEGs, this molecule is not part of the dimer interface.

The effect of crowding agents in 3D-DS has been studied as it increases local protein concentration and facilitates protein oligomerization (Liu & Eisenberg, 2002 ▸; Mateos *et al.*, 2021 ▸). Previous studies have shown that, as a macromolecular crowding agent, PEG has different effects on protein domain swapping depending on the system studied and can have both promoting and inhibiting effects under specific conditions. Besides, the effect of PEG on the structure and conformational stability of 3D-DS depends on its size and concentration (Stepanenko *et al.*, 2016 ▸). Crowding agents cause compaction by excluding volume, where the compact hydrophobic core of the protein remains unaffected, and most of the changes are expected to occur in loops and turns on the surface of the protein. However, the interaction of PEG with proteins cannot be described quantitatively in terms of excluded volume alone. In the case of low-molecular-mass PEGs, interactions at the open interface or with solvent-exposed residues would also energetically favour the formation of the intertwined dimer.

## 5. Conclusion

Local unfolding events can give rise to near-native intermediates whose role in 3D-DS has been studied using structure-based model simulations of the γ-crystallins (Das *et al.*, 2011 ▸) and SH3 domains (Ding *et al.*, 2005 ▸; Zhuravlev *et al.*, 2014 ▸). These simulations indicated the presence of an open monomer in the folding pathway which, under certain conditions, can form a tightly packed hydrophobic core by either forming a monomer or an intertwined dimer. As the closed interface is conserved, new interactions in the open interface and lower conformational energy might favour the intertwined oligomer (Ding *et al.*, 2002 ▸). Furthermore, transient interactions can also displace the folding equilibrium to monomer or dimer formation (Assar *et al.*, 2016 ▸).

The folding mechanism of the SH3 domain has been extensively characterized using experimental approaches and molecular-dynamics computations (Grantcharova & Baker, 1997 ▸; Riddle *et al.*, 1997 ▸, 1999 ▸; Grantcharova *et al.*, 1998 ▸, 2000 ▸; Ding *et al.*, 2002 ▸, 2005 ▸; Klimov & Thirumalai, 2002 ▸; Martinez & Serrano, 1999 ▸; Martinez *et al.*, 1998 ▸). In c-Src, as happens in other members of this domain, the native structure of the diverging β-turn is present early in the folding reaction because its formation is not dependent on interactions with other parts of the domain, which may fold later (Grantcharova *et al.*, 2000 ▸). Meanwhile, the interaction of the RT loop with the central three-stranded β-sheet is facilitated by hydrophobic contacts between the core residues, Phe102 and Leu108, and a hydrogen bond between the side chain of Glu106 and the backbone N atom of Lys103. The weak interactions between the folding nucleus and the RT loop may explain its late folding, which facilitates the exchange of this loop in the c-Src SH3 domain to form intertwined dimers under pH and concentration conditions that facilitate oligomerization. Previous folding studies have reported that the two terminal β-strands, the RT loop and the 3_10_-helix are mainly unstructured and contribute through a few stabilizing interactions in the transition state (Riddle *et al.*, 1999 ▸). Moreover, clustering mutations near the n-Src loop that selectively stabilize or destabilize the transition state suggest that the loop may have a non-native conformation in the transition state (Riddle *et al.*, 1999 ▸). This non-native conformation would be compatible with the role of the n-Src loop as a hinge loop.

Our results agree with the hypothesis that domain swapping occurs in regions where the protein tends to unfold before complete unfolding (Ding *et al.*, 2006 ▸). The formation of the open monomer may be the rate-limiting step, as forming domain-swapped oligomers from open monomers is not an energetically demanding process: an open monomer and a domain-swapped dimer share the same structure except for the hinge region. In this way, such secondary elements, which are present at the early stages of folding, must already be present in the domain-swapped structures. The results obtained in this work suggest that although hinge loops play a crucial role in the domain-swapping event of the SH3 domain, new interactions in the open interface of the oligomer must also be considered. Moreover, as in all oligomerization processes, the formation of intertwined dimers is dependent on protein concentration. Those circumstances that reduce protein solubility also impair the formation of intertwined dimers. Finally, an in-depth analysis of domain-swapped structures could be a powerful tool for studying long-range interactions *in vitro* that participate in the protein-folding process and secondary-structure elements already present in the early folding stages.

## Supplementary Material

PDB reference: Abl_Src-2, monomer, 7pvq

PDB reference: Abl_Src-2, monomer, 7pvs

PDB reference: Abl_Src-nSrc, monomer, 7pvv

PDB reference: Src_Abl-2, intertwined, 7pvw

PDB reference: Src_Abl-RT, monomer, 7pvy

PDB reference: Src_Abl-RT, intertwined, 7pvz

PDB reference: Src_Abl-nSrc, monomer, 7pw0

PDB reference: Abl_Src-RT, monomer, 7pw2

Supplementary Figures and Table,. DOI: 10.1107/S2059798325006977/ud5058sup1.pdf

## Figures and Tables

**Table:** 

	Src_Abl-RT	Src_Abl-RT	Src_Abl-nSrc	Src_Abl-2
PDB code	7pvy	7pvz	7pw0	7pvw
Method	Vapour diffusion, sitting drop	Vapour diffusion, sitting drop	Vapour diffusion, sitting drop	Vapour diffusion, sitting drop
Plate type	48-well protein-crystallization plate	24-well protein-crystallization plate	48-well protein-crystallization plate	48-well protein-crystallization plate
Temperature (K)	298	298	277	298
Protein concentration (mg ml^−1^)	10	10	4	10
Buffer composition of protein solution	10 m*M* Tris–HCl pH 8.0	5% PEG 300, 10 m*M* acetic acid/sodium acetate pH 5.0	10 m*M* Tris–HCl pH 8.0	5% PEG 300, 10 m*M* acetic acid/sodium acetate pH 5.0
Composition of reservoir solution	1.5 *M* ammonium sulfate, 5% PEG 4000, 0.1 *M* Tris–HCl pH 8.0	1.5 *M* ammonium sulfate, 5% PEG 300, 0.1 *M* acetic acid/sodium acetate pH 5.0	1.8 *M* ammonium sulfate, 5% PEG 300, 10% glycerol, 40 m*M* lithium chloride, 0.1 *M* acetic acid/sodium acetate pH 5.5	0.9 *M* ammonium sulfate, 0.1 *M* acetic acid/sodium acetate pH 4.0
Volume and ratio of drop	3 µl:3 µl	3 µl:3 µl	2 µl:2 µl	2 µl:2 µl
Volume of reservoir (µl)	200	1000	200	200

**Table:** 

	Abl_Src-RT	Abl_Src-nSrc	Abl_Src-2	Abl_Src-2
PDB code	7pw2	7pvv	7pvq	7pvs
Method	Vapour diffusion, sitting drop	Vapour diffusion, sitting drop	Vapour diffusion, sitting drop	Vapour diffusion, sitting drop
Plate type	48-well protein-crystallization plate	48-well protein-crystallization plate	48-well protein-crystallization plate	48-well protein-crystallization plate
Temperature (K)	298	283	288	283
Protein concentration (mg ml^−1^)	8	8	10	10
Buffer composition of protein solution	10 m*M* Tris–HCl pH 8.0	10% glycerol, 40 m*M* lithium chloride, 10 m*M* Tris–HCl pH 8.0	10% glycerol, 40 m*M* lithium chloride, 10 m*M* Tris–HCl pH 8.0	10% glycerol, 40 m*M* lithium chloride, 10 m*M* Tris–HCl pH 8.0
Composition of reservoir solution	2.0 *M* ammonium sulfate, 0.1 *M* HEPES–NaOH pH 7.0	1.6 *M* ammonium sulfate, 5% PEG 200, 10% glycerol, 40 m*M* lithium chloride, 0.1 *M* acetic acid/sodium acetate pH 5.5	1.8 *M* ammonium sulfate, 0.1 *M* MES–NaOH pH 6.0	2.6 *M* ammonium sulfate, 5% PEG 200, 10% glycerol, 40 m*M* lithium chloride, 0.1 *M* MES–NaOH pH 6.5
Volume and ratio of drop	3 µl:3 µl	2 µl:2 µl	2 µl:2 µl	3 µl:3 µl
Volume of reservoir (µl)	200	200	200	200

**Table:** Values in parentheses are for the outer shell.

	Src_Abl-RT, intertwined	Src_Abl-2, intertwined	Src_Abl-nSrc, monomer	Src_Abl-RT, monomer
PDB code	7pvz	7pvw	7pw0	7pvy
Diffraction source	BL13 XALOC, ALBA	BL13 XALOC, ALBA	MASSIF-3, ESRF	BL13 XALOC, ALBA
Wavelength (Å)	0.98	0.98	0.98	0.98
Temperature (K)	100	100	100	100
Detector	Dectris PILATUS 6M	Dectris PILATUS 6M	Dectris EIGER X 4M	Dectris PILATUS 6M
Crystal-to-detector distance (mm)	389	242	125	267
Rotation range per image (°)	0.20	0.15	0.20	0.15
Total rotation range (°)	360	270	220	290
Space group	*P*6_4_	*P*6_3_	*P*2_1_	*P*22_1_2_1_
*a*, *b*, *c* (Å)	48.8, 48.8, 90.9	68.0, 68.0, 46.2	37.3, 93.1, 80.4	26.0, 41.5, 52.2
α, β, γ (°)	90, 90, 90	90, 90, 90	90, 97.3, 90	90, 90, 90
Mosaicity† (°)	0.143	0.150	0.204	0.157
Resolution range‡ (Å)	19.16–2.00 (2.05–2.00)	19.64–1.50 (1.53–1.50)	19.95–1.70 (1.73–1.70)	19.28–1.40 (1.42–1.40)
Total No. of reflections	83456 (6392)	126662 (6481)	255084 (13079)	105356 (3536)
No. of unique reflections	8310 (833)	19606 (1963)	59211 (3116)	13544 (512)
Completeness (%)	99.9 (100.0)	99.9 (100.0)	99.0 (98.7)	98.5 (96.6)
Multiplicity	10.0 (10.3)	6.5 (6.6)	4.3 (4.2)	9.2 (6.4)
Average *I*/σ(*I*)§	24.2 (3.6)	17.9 (2.3)	9.4 (1.1)	16.5 (2.4)
CC_1/2_	0.998 (0.866)	0.999 (0.838)	0.998 (0.542)	0.997 (0.956)
*R*_merge_	0.04 (0.63)	0.04 (0.95)	0.06 (1.15)	0.06 (0.52)
Overall *B* factor from Wilson plot (Å^2^)	53	23	23	19
Average *B* factor (Å^2^)	68	33	36	30

**Table:** 

	Abl_Src-RT, monomer	Abl_Src-nSrc, monomer	Abl_Src-2, monomer	Abl_Src-2, monomer
PDB code	7pw2	7pvv	7pvq	7pvs
Diffraction source	BL13 XALOC, ALBA	BL13 XALOC, ALBA	BL13 XALOC, ALBA	BL13 XALOC, ALBA
Wavelength (Å)	0.98	0.98	0.98	0.98
Temperature (K)	100	100	100	100
Detector	Dectris PILATUS 6M	Dectris PILATUS 6M	Dectris PILATUS 6M	Dectris PILATUS 6M
Crystal-to-detector distance (mm)	159	369	308	160
Rotation range per image (°)	0.10	0.15	0.10	0.10
Total rotation range (°)	180	290	170	150
Space group	*P*4_1_	*I*2_1_2_1_2_1_	*P*2_1_22_1_	*P*2_1_22_1_
*a*, *b*, *c* (Å)	42.6, 42.6, 30.4	41.9, 47.4, 75.6	27.3, 43.5, 94.8	27.1, 43.5, 94.6
α, β, γ (°)	90, 90, 90	90, 90, 90	90, 90, 90	90, 90, 90
Mosaicity† (°)	0.111	0.088	0.196	0.139
Resolution range‡ (Å)	17.44–1.10 (1.12–1.10)	19.90–1.82 (1.86–1.82)	19.76–1.55 (1.58–1.55)	19.75–1.05 (1.07–1.05)
Total No. of reflections	43012 (2134)	33491 (1728)	41512 (1278)	271721 (8357)
No. of unique reflections	21188 (2155)	7057 (343)	15097 (1488)	52808 (4975)
Completeness (%)	95.3 (97.0)	98.9 (89.8)	90.1 (89.2)	99.3 (92.9)
Multiplicity	2.0 (2.0)	4.6 (3.2)	2.7 (1.8)	5.1 (3.5)
Average *I*/σ(*I*)§	9.9 (1.6)	13.3 (1.3)	13.3 (3.9)	14.8 (1.9)
CC_1/2_	0.998 (0.683)	0.999 (0.735)	0.992 (0.915)	1.000 (0.760)
*R*_merge_	0.02 (0.42)	0.04 (0.56)	0.06 (0.26)	0.04 (0.61)
Overall *B* factor from Wilson plot (Å^2^)	18	28	13	10
Average *B* factor (Å^2^)	28	37	18	14

† As defined in *XDS* (Kabsch, 2010 ▸).

‡ The resolution cutoff was determined based on the CC_1/2_ criterion (Karplus & Diederichs, 2012 ▸).

§ Average *I*/σ(*I*), as defined in *AIMLESS*.

**Table:** Values in parentheses are for the outer shell.

	Src_Abl-RT, intertwined	Src_Abl-2, intertwined	Src_Abl-nSrc, monomer	Src_Abl-RT, monomer
PDB code	7pvz	7pvw	7pw0	7pvy
Resolution range (Å)	19.16–2.00 (2.13–2.00)	19.64–1.50 (1.53–1.50)	19.95–1.70 (1.72–1.70)	19.28–1.40 (1.47–1.40)
Completeness (%)	99.9 (100.0)	99.9 (100.0)	97.5 (100.0)	97.9 (100.0
No. of reflections, working set	15501 (2555)	36119 (2540)	109274 (3538)	19996 (2884)
No. of reflections, test set	843 (185)	1939 (162)	5685 (193)	1040 (125)
Final *R*_cryst_	0.23 (0.36)	0.16 (0.23)	0.21 (0.42)	0.20 (0.28)
Final *R*_free_	0.26 (0.35)	0.19 (0.26)	0.23 (0.40)	0.22 (0.33)
No. of non-H atoms				
Protein	893	929	3624	432
Ion	0	4	0	0
Ligand	0	13	0	0
Water	3	85	386	49
Total	896	1052	4010	481
R.m.s. deviations				
Bond lengths (Å)	0.013	0.014	0.007	0.009
Angles (°)	1.47	1.32	1.14	1.27
Average *B* factors (Å^2^)				
Protein	61.2	28.9	33.1	26.3
Ion	0	54.4	0	0
Ligand	0	52.2	0	0
Water	86	41.1	39.4	40.9
Ramachandran plot				
Most favoured (%)	100.00	100.00	96.85	100.00
Allowed (%)	0.00	0.00	3.15	0.00
Rotamer outliers (%)	1.15	0.00	0.27	0.00
Clashcore	1.17	0.54	1.30	0.00

**Table:** 

	Abl_Src-RT, monomer	Abl_Src-nSrc, monomer	Abl_Src-2, monomer	Abl_Src-2, monomer
PDB code	7pw2	7pvv	7pvq	7pvs
Resolution range (Å)	17.44–1.10 (1.13–1.10)	19.90–1.82 (2.29–1.82)	19.76–1.55 (1.61–1.55)	19.75–1.05 (1.06–1.05)
Completeness (%)	75.4	99.2	79.9	98.1
No. of reflections, working set	30963 (2412)	12317 (3140)	24092 (2459)	93949 (2629)
No. of reflections, test set	1752 (152)	610 (146)	1203 (106)	5061 (138)
Final *R*_cryst_	0.183 (0.279)	0.210 (0.236)	0.231 (0.2705)	0.169 (0.2580)
Final *R*_free_	0.201 (0.327)	0.224 (0.275)	0.274 (0.2916)	0.184 (0.2722)
No. of non-H atoms				
Protein	421	432	914	934
Ion	0	0	0	16
Ligand	0	17	0	36
Water	51	40	147	141
Total	472	513	1061	1177
R.m.s. deviations				
Bond lengths (Å)	0.017	0.006	0.010	0.011
Angles (°)	1.66	1.02	1.23	1.44
Average *B* factors (Å^2^)				
Protein	24.8	37.3	15.4	11.9
Ion	0	0	0	30.0
Ligand	0	38.6	0	27.1
Water	36.9	41.5	25.4	23.4
Ramachandran plot				
Most favoured (%)	96.36	96.36	96.43	99.12
Allowed (%)	3.64	3.64	3.57	0.88
Rotamer outliers (%)	2.38	0.00	1.98	1.82
Clashcore	0.00	0.00	0.00	3.00

**Table 4:** Apparent p*K*_a_ values of acid and basic denaturation of the Src_Abl and Abl_Src chimeras

			No. of ionizable residues						
	p*K*_a_ (acid)	p*K*_a_ (basic)	Asp	Glu	His	Tyr	Lys	Arg	Cys
Src_Abl-RT	4.0 ± 0.1	10.9 ± 0.1	4	3	1	4	2	1	0
Src_Abl-nSrc	4.2 ± 0.1	10.7 ± 0.1	4	3	2	5	2	2	0
Src_Abl-2	4.0 ± 0.1	10.8 ± 0.1	4	2	2	5	2	1	0
Abl_Src-RT	4.7 ± 0.1	9.4 ± 0.1	1	5	1	3	3	2	1
Abl_Src-nSrc	4.3 ± 0.1	9.7 ± 0.1	2	4	0	2	3	1	1
Abl_Src-2	4.4 ± 0.1	9.3 ± 0.1	1	6	0	2	3	2	1

**Table 5:** Thermodynamic parameters of the Src_Abl and Abl_Src chimeras obtained by chemical denaturation with GdnHCl

	*D*_1/2_ (*M*)	Δ*G*_w_ (kJ mol^−1^)
Src_Abl-RT	3.2 ± 0.3	15 ± 1
Src_Abl-nSrc	2.7 ± 0.3	15 ± 2
Src_Abl-2	3.5 ± 0.4	18 ± 1
Abl_Src-RT	2.8 ± 0.3	14 ± 2
Abl_Src-nSrc	1.5 ± 0.2	10 ± 2
Abl_Src-2	1.0 ± 0.1	10 ± 2

**Table 6:** Apparent thermodynamic parameters of the thermal denaturation of the Src and Abl SH3 chimeras

	*T*_m_ (K)	Δ*H*_m_ (kJ mol^−1^)	Δ*G* (kJ mol^−1^)	−*T*Δ*S* (kJ mol^−1^)
Src_Abl-RT	347.0 ± 0.2	268 ± 11	26 ± 3	−81 ± 22
Src_Abl-nSrc	347.0 ± 0.2	224 ± 6	20 ± 2	−43 ± 21
Src_Abl-2	347.4 ± 0.9	232 ± 17	20 ± 2	−49 ± 26
c-Src WT†	348.6 ± 0.5	321 ± 32	34 ± 4	−121 ± 38
Abl_Src-RT	334.3 ± 0.1	197 ± 3	15 ± 2	−63 ± 15
Abl_Src-nSrc	319.0 ± 0.4	116 ± 6	6 ± 1	−41 ± 10
Abl_Src-2	313.0 ± 1.3	87 ± 11	3 ± 1	−35 ± 13
Abl-WT‡	341.5 ± 0.2	194 ± 10	15 ± 2	−36 ± 20

† Plaza-Garrido *et al.* (2020 ▸).

‡ Δ*C*_p_ = 3.3 kJ K^−1^ mol^−1^ (Filimonov *et al.*, 1999 ▸).
