# Supplementary material for: Understanding domain swapping in the c-Src SH3 domain through hinge-loop mutagenesis
Source: Acta Crystallogr D Struct Biol. 2025 Aug 27;81(Pt 9):492–510. doi: 10.1107/S2059798325006977 (PMC12400191; doi:10.1107/S2059798325006977)
Supplement: Supplementary file 1 [file d-81-00492-sup1.pdf]

STRUCTURAL
BIOLOGY

Volume 81 (2025)

Supporting information for article:

Understanding domain swapping in the c-Src SH3 domain through hinge-loop mutagenesis

M. Carmen Salinas-Garcia, Marina Plaza-Garrido, Jose C. Martinez and Ana Camara-Artigas

Figure S1 (A) Fluorescence spectra collected to determine the stability of the Src_Abl and Abl_Src chimeras *vs* pH. The samples were excited at 280 nm, and the fluorescence intensity was measured at 350 nm, the maximum of the emission spectrum, as described in the Materials and Methods section.

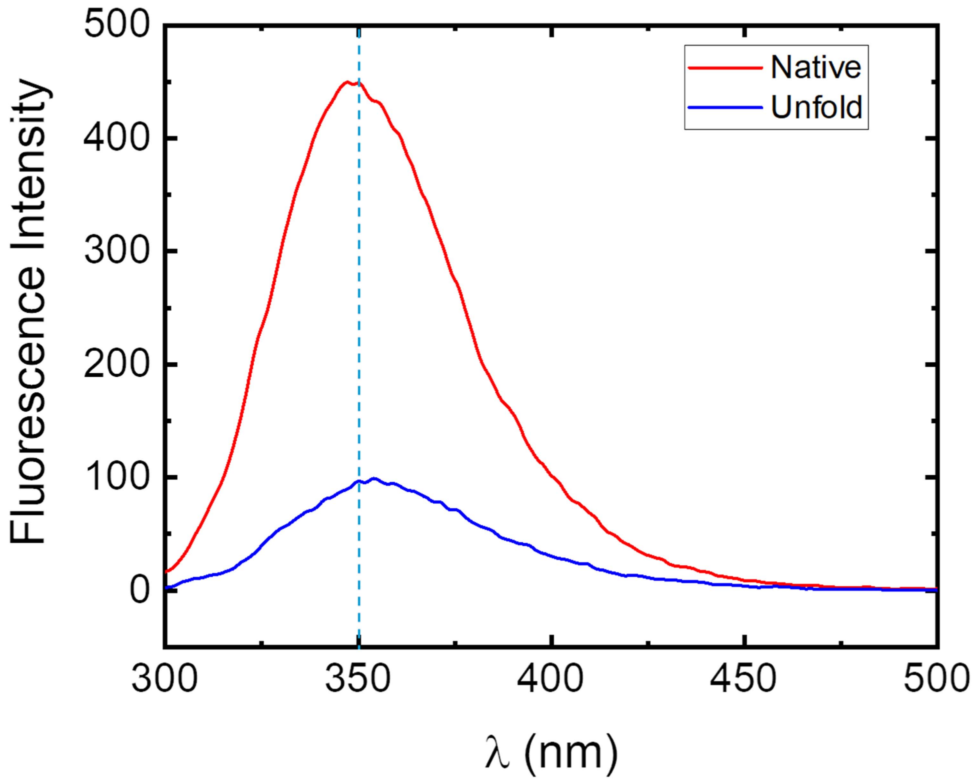

(B) Fluorescence spectra collected to determine the stability of the Src_Abl and Abl_Src chimeras vs guanidium hydrochloride concentration. In these experiments, as can be seen in the figure, the wavelength of the maximum of the emission spectra is, to some extent, shifted to the red. The wavelength-averaged emission intensity $\bar{Y}$ was calculated using Eq. 4.

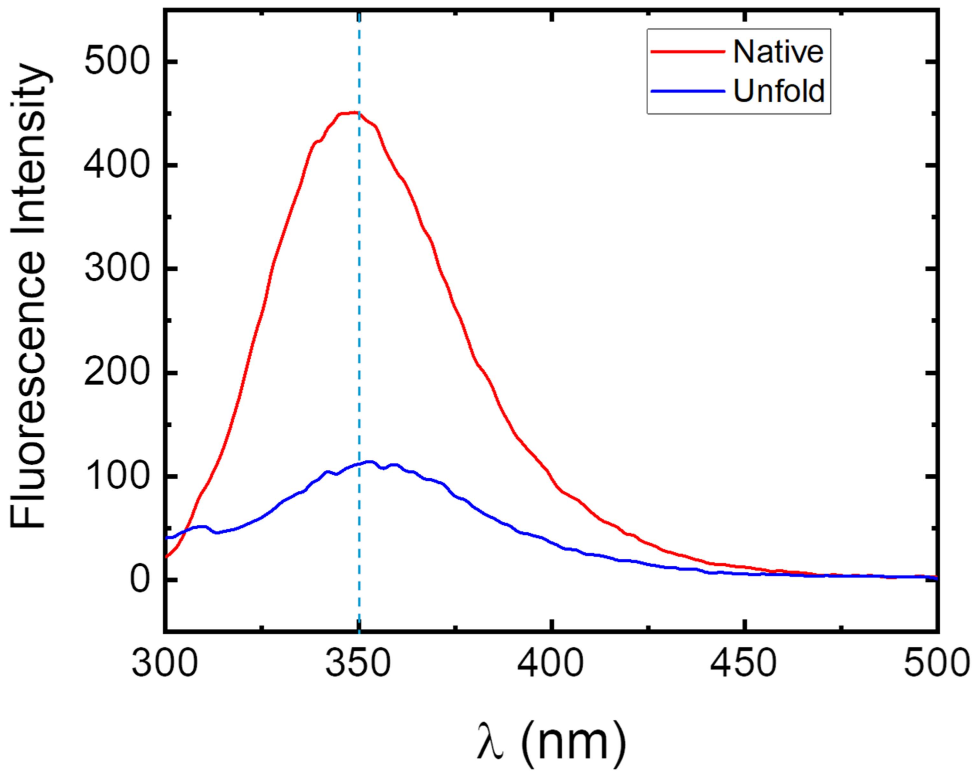

Figure S2 (A) CD spectra of the native and the unfolded state of the Src_Abl. Thermal denaturation was performed using a constant heating rate of 120 °C/h from 10 to 95 °C, based on the change in ellipticity at 223 nm, which was determined by the maximum difference between the spectra of the native and unfolded chimaeras, while maintaining the voltage in a lower noise range.

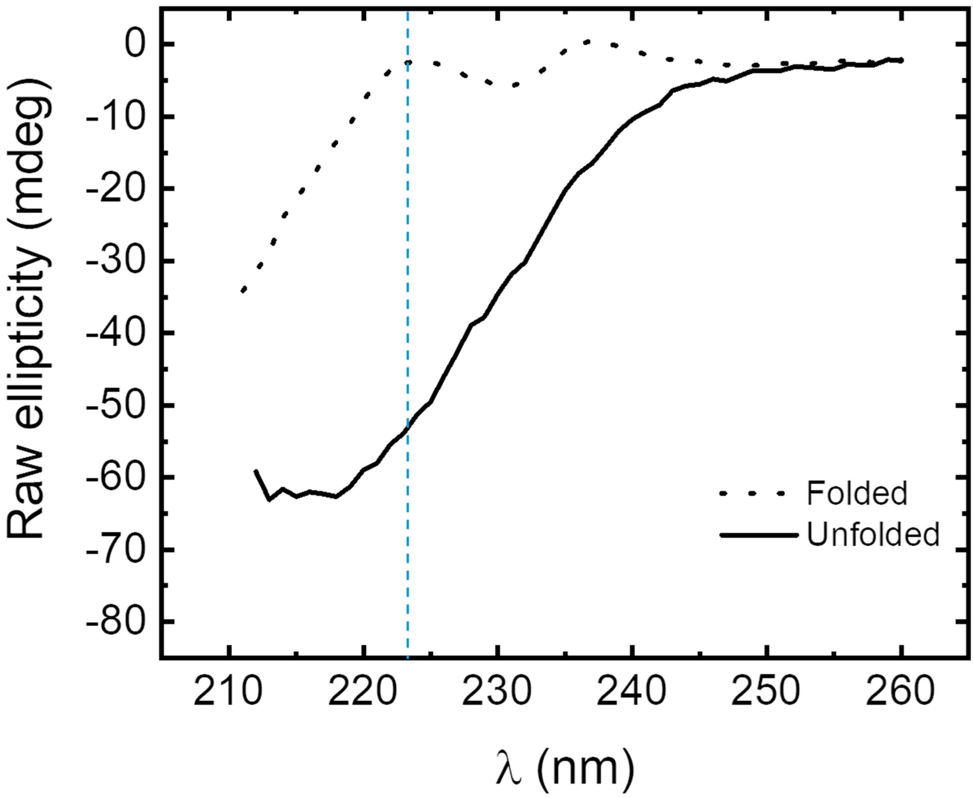

(B) CD spectra of the native and the unfolded state of the Abl_Src. Thermal denaturation was performed using a constant heating rate of 120 °C/h from 10 to 95 °C, as indicated by the change in ellipticity at 230 nm, based on the maximum difference between the spectra of the native and unfolded chimaeras, while maintaining the voltage in a lower noise range.

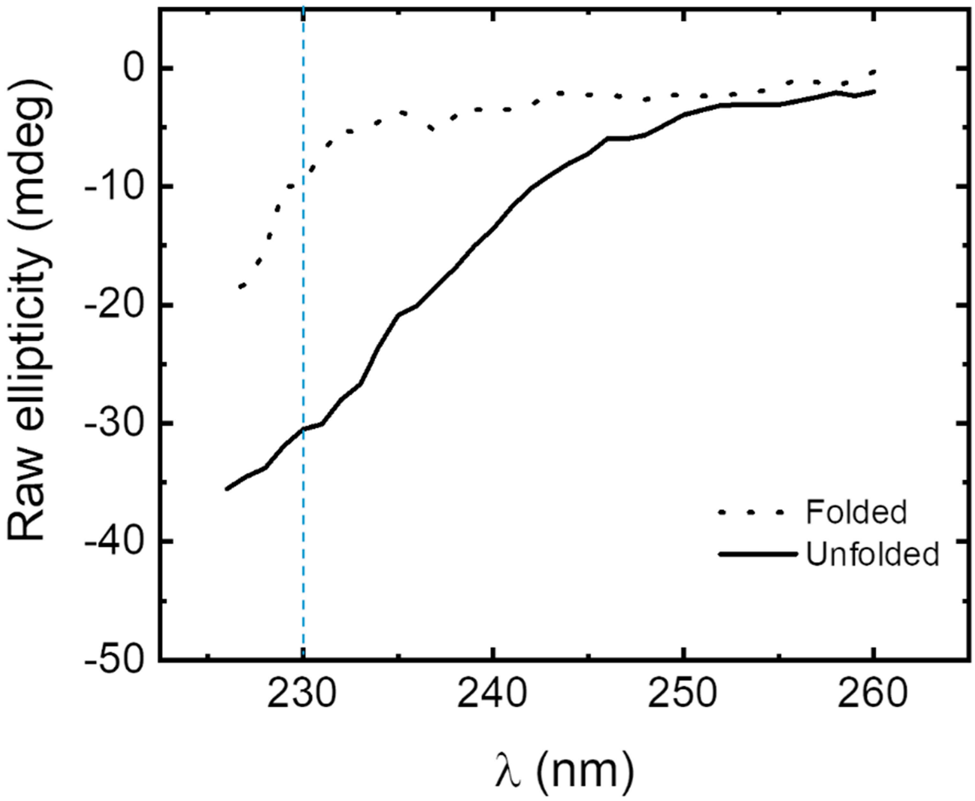

Figure S3 Crystals of the Abl_Src-nSrc obtained using 1.6 M ammonium sulfate, 5% PEG 200, 10% glycerol, 40 mM lithium chloride and 0.1M acetic acid /sodium acetate (pH 5.5) as precipitant solution. Crystals were improved using seeding techniques, as described in the Materials and Methods section. A) The red narrow marks the small crystals obtained before applying seeding techniques. B) Crystals improved using seeding techniques.

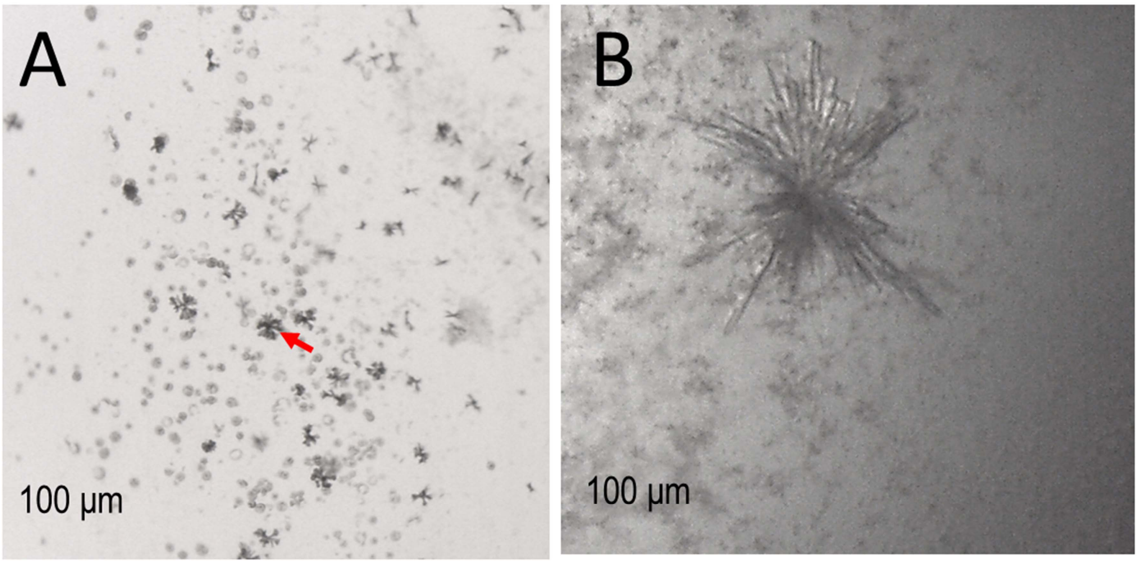

Figure S4 2D topology diagrams of the monomeric structure of (A) Src_Abl-nSrc, and the intertwined dimers of (B) Src_Abl-2, (C) Src_Abl-RT, and (D) WT

(A)

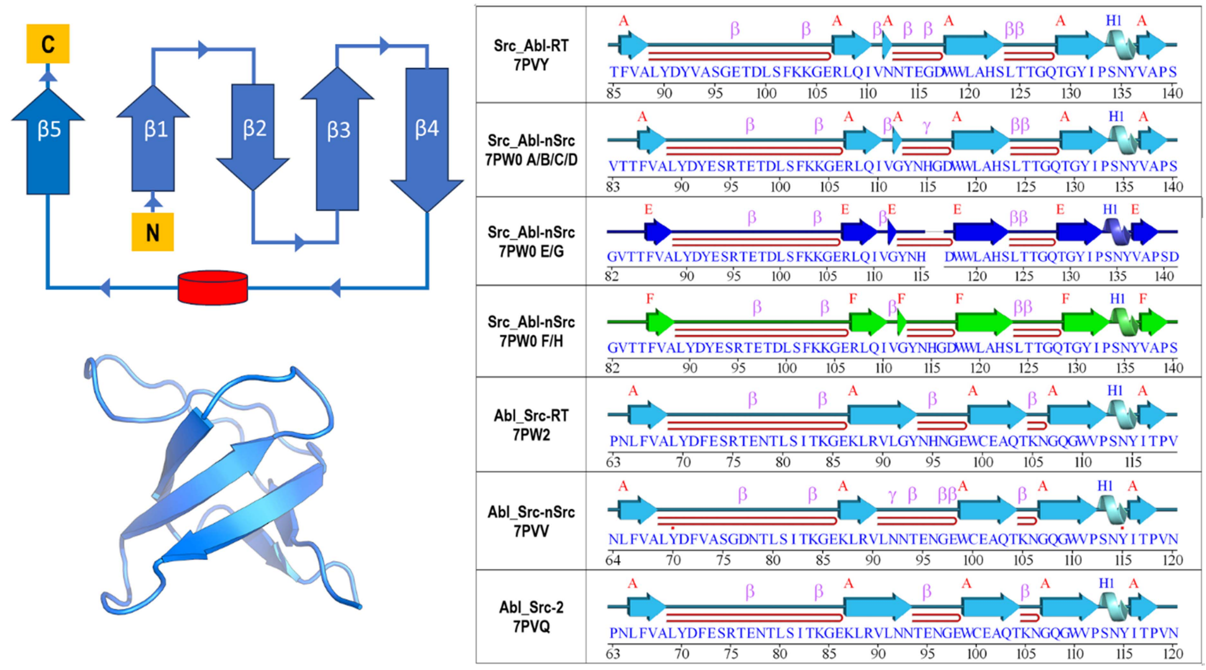

(B)

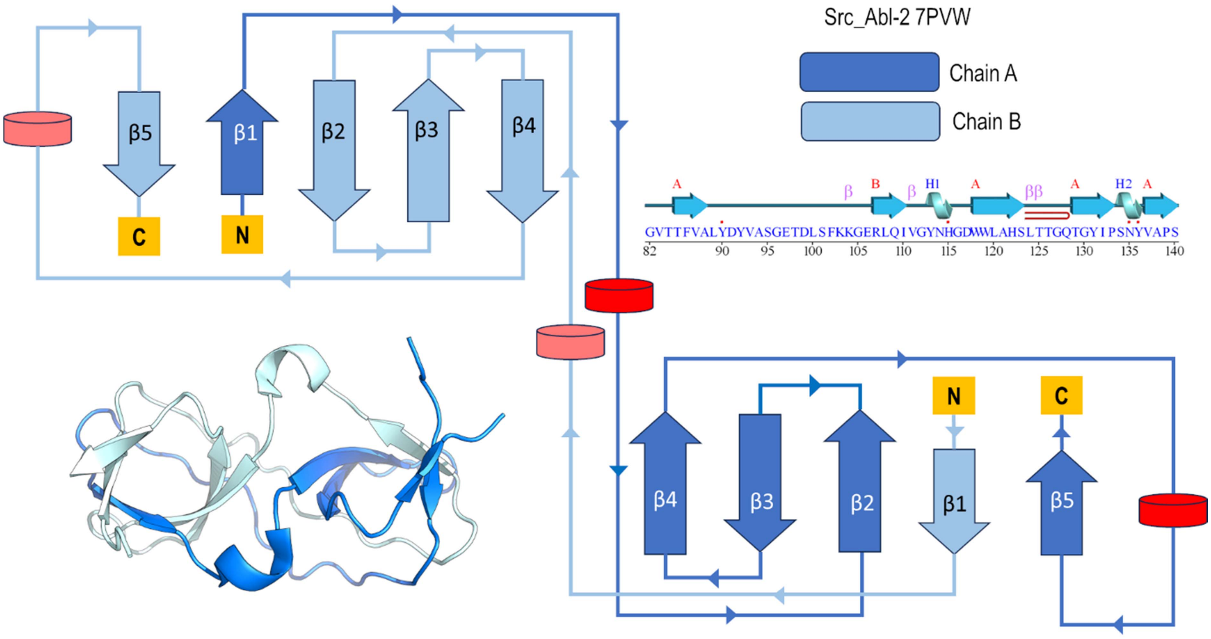

(C)

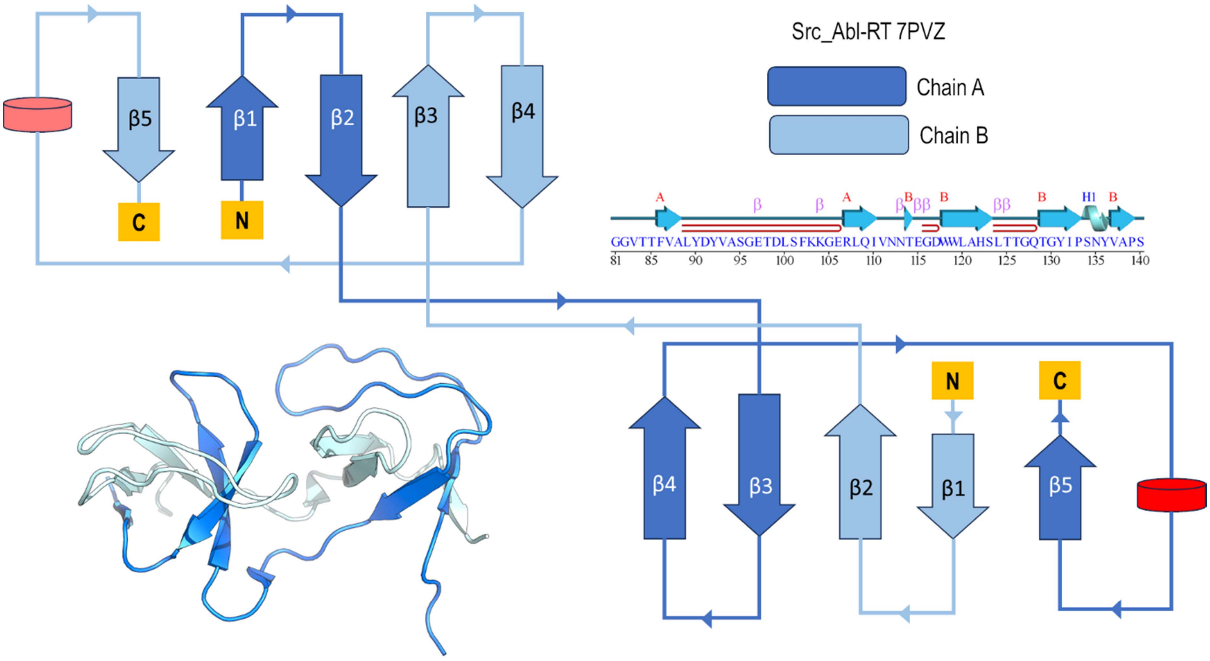
Src_Abl-RT 7PVZ
Chain A
Chain B
β5
β1
β2
β3
β4
C
N
GGVTTFVALYDYVASGETDLSFKKGERLQIVNNTEGDWWLAHSLTTGQTGYIPSNYVAPS
81 85 90 95 100 105 110 115 120 125 130 135 140
β4
β3
β2
β1
β5
N
C

(D)

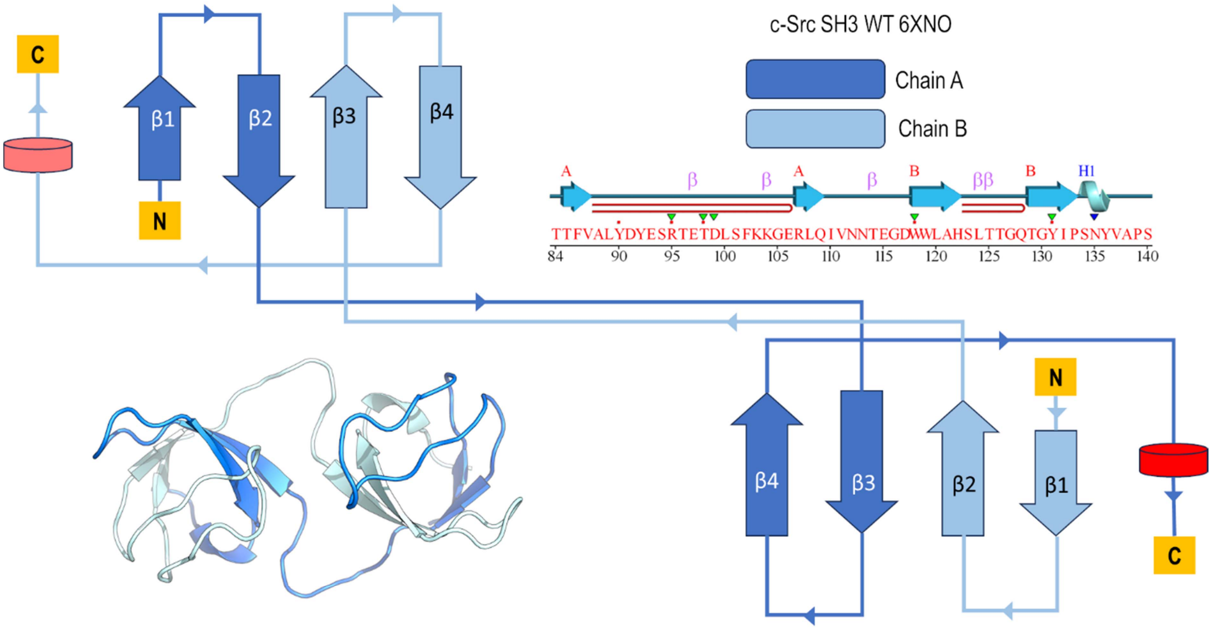
c-Src SH3 WT 6XNO
Chain A
Chain B
C
N
β1
β2
β3
β4
TTFVALYDYESRTETDLSFKKGERLQIVNNTEGDWWLAHSLTTGQTGYIPSNYVAPS
84 90 95 100 105 110 115 120 125 130 135 140
β4
β3
β2
β1
N
C

Table S1 Solubility of the Src_Abl and Abl_Src chimeras. The solubility of the chimeras was assayed using the lyophilised method of Trevino et al. (Trevino et al., 2008) at two different pHs: pH 5, 50 mM acetic acid/sodium acetate, and; pH 7, 50 mM sodium phosphate monobasic/dibasic.

	Solubility (mg/mL)	
	pH 5.0	pH 7.0
Src_Abl-RT	14.8±0.4	13.7±0.2
Src_Abl-nSrc	4.5±0.2	4.8±0.3
Src_Abl-2	10.4±0.3	12.5±0.2
Abl_Src-RT	4.0±0.2	10.0±1.3
Abl_Src-nSrc	5.2±0.1	11.0±0.6
Abl_Src-2	2.8±0.2	3.8±0.2
